# Extracellular vesicles derived from SARS-CoV-2 M-protein-induced triple negative breast cancer cells promoted the ability of tissue stem cells supporting cancer progression

**DOI:** 10.3389/fonc.2024.1346312

**Published:** 2024-03-07

**Authors:** Hoai-Nga Thi Nguyen, Cat-Khanh Vuong, Mizuho Fukushige, Momoko Usuda, Liora Kaho Takagi, Toshiharu Yamashita, Mana Obata-Yasuoka, Hiromi Hamada, Motoo Osaka, Toru Tsukada, Yuji Hiramatsu, Osamu Ohneda

**Affiliations:** ^1^ Laboratory of Regenerative Medicine and Stem Cell Biology, Graduate School of Comprehensive Human Science, University of Tsukuba, Tsukuba, Japan; ^2^ Department of Obstetrics and Gynecology, University of Tsukuba, Tsukuba, Japan; ^3^ Department of Cardiovascular Surgery, University of Tsukuba, Tsukuba, Japan

**Keywords:** SARS-CoV-2, breast cancer, extracellular vesicles, tissue stem cells, tumor microenvironment

## Abstract

**Introduction:**

SARS-CoV-2 infection increases the risk of worse outcomes in cancer patients, including those with breast cancer. Our previous study reported that the SARS-CoV-2 membrane protein (M-protein) promotes the malignant transformation of triple-negative breast cancer cells (triple-negative BCC).

**Methods:**

In the present study, the effects of M-protein on the ability of extracellular vesicles (EV) derived from triple-negative BCC to regulate the functions of tissue stem cells facilitating the tumor microenvironment were examined.

**Results:**

Our results showed that EV derived from M-protein-induced triple-negative BCC (MpEV) significantly induced the paracrine effects of adipose tissue-derived mesenchymal stem cells (ATMSC) on non-aggressive BCC, promoting the migration, stemness phenotypes, and *in vivo* metastasis of BCC, which is related to PGE2/IL1 signaling pathways, in comparison to EV derived from normal triple-negative BCC (nEV). In addition to ATMSC, the effects of MpEV on endothelial progenitor cells (EPC), another type of tissue stem cells, were examined. Our data suggested that EPC uptaking MpEV acquired a tumor endothelial cell-like phenotype, with increasing angiogenesis and the ability to support the aggressiveness and metastasis of non-aggressive BCC.

**Discussion:**

Taken together, our findings suggest the role of SARS-CoV-2 M-protein in altering the cellular communication between cancer cells and other non-cancer cells inside the tumor microenvironment via EV. Specifically, M-proteins induced the ability of EV derived from triple-negative BCC to promote the functions of non-cancer cells, such as tissue stem cells, in tumorigenesis.

## Introduction

In the coronavirus disease 2019 (COVID-19) pandemic, cancer patients have the increased SARS-CoV-2 incidences and become more vulnerable to SARS-CoV-2 infection, according to previous pan-cancer studies (1, 2). Among cancer patients, those with breast cancer patients show high expression of TMPRSS2, a SARS-CoV-2 infection-associated gene and have poor prognosis prediction (3). In addition, breast cancer patients with COVID-19 have a significant increase in serum cancer biomarker levels, suggesting the influence of SARS-CoV-2 infection on tumorigenesis (4–6). Numerous studies suggested a high risk of worse outcomes in breast cancer patients with COVID-19 due to systemic inflammatory cytokine storms (7–11) and the increased malignancy of cancer cells, resulting in new metastasis, progress and death (5, 6, 12, 13).

Although the COVID-19 pandemic has been controlled, the long-term post-COVID-19 syndrome still causes many concerns for patients with cancer, including breast cancer. After recovery from COVID-19, SARS-CoV-2 viral proteins can be detected in patient sera (14–17); therefore, the effects of SARS-CoV-2 proteins on cancer cells and the tumor microenvironment (TME), which support the development of tumors, should be studied. We previously reported that the SARS-CoV-2 membrane protein (M-protein) promotes the malignancy of triple-negative BCC (6). However, whether M-protein-induced triple-negative BCC show altered behaviors in regulating non-cancer cells inside the TME remains obscure.

In the TME, cellular communication is performed by both direct cell-cell contact and classical paracrine signals, in which extracellular vesicles (EV) are a crucial means of communication (18). EV are lipid bilayer-bound vehicles secreted from the cell membrane, and their contents reflect the original cell, including signaling proteins, RNA, and DNA (18). Cancer cells constantly produce and release EV into the extracellular space, which transmits information to surrounding cells and even distant target cells (18, 19). EV facilitate specific cell-cell interactions and stimulate signaling pathways in their target cells (18) to support tumor development (20, 21). Notably, previous studies have suggested that the functions of cancer cell-derived EV in the TME are associated with the aggressiveness of parental cancer cells. For instance, breast cancer cells with oncogene overexpression altered their EV content toward a malignant phenotype (22, 23). Although many studies have reported the effects of SARS-CoV-2 proteins on cancer cells, its effects on EV derived from cancer cells remain obscure.

The TME consists of cancer and non-cancer cells, including fibroblast, immune cells, endothelial cells and tissue stem cells such as mesenchymal stem cells (MSC) and endothelial progenitor cells (EPC) (19, 24). We previously reported the abilities of MSC derived from adipose tissues to support the metastasis of triple negative BCC (25). In addition, previous studies reported that adapting to cancer signals in the TME, MSC change their behavior and synergize with cancer cells (26, 27). MSC can differentiate into cancer-associated fibroblasts or evolve into tumor-associated mesenchymal stem cells (TA-MSC) under the control of local factors in the TME (28, 29). However, up to date the effects of SARS-CoV-2 proteins on the breast cancer signals regulating MSC are still obscured.

Another type of tissue stem cells involved in tumor microenvironment besides MSC is EPC, which belong to endothelial lineage cells (30). EPC are recruited toward tumors from the blood by signals from cancer cells and contribute to tumor growth and angiogenesis, thereby transporting nutrients to the tumor core and maintaining metabolic homeostasis (31–33). In addition, the loose cell-cell connections inside blood vessels formed in the tumor facilitates the intravasation of circulating cancer cells, which initiates the process of metastasis (24). Moreover, in the TME, EPC differentiate into tumor endothelial cells (TEC) (34), which form new blood vessels and produce cytokines and growth factors for tumor growth and invasion (35). Notably, tumor angiogenesis in EPC and other endothelial lineage cells is strongly supported by MSC located inside the tumor microenvironment (26, 28).

In the present study, we examined the effects of the SARS-CoV-2 M-protein on the ability of EV derived from triple-negative BCC to regulate the characteristics and tumorigenic functions of tissue stem cells, including MSC and EPC.

## Results

### EV derived from M-protein-induced triple-negative BCC increased the cancer-supporting gene expression of ATMSC but did not alter their features

Breast tumors are mainly surrounded by mammary adipose tissue and merge with a repertoire of MSC, which interact mutually with cancer cells (36). In the TME, MSC derived from adipose tissues (ATMSC) receive stimulation signals from BCC to evolve into tumor-associated mesenchymal stem cells (TA-MSC) or to differentiate into cancer-associated fibroblasts (22, 28, 29). In addition, our previous study reported the ability of ATMSC inducing the metastasis of BCC. Therefore, we first examined the effects of M-protein on the ability of EV derived from triple-negative BCC to regulate ATMSC. EV were isolated from the conditioned medium of original triple-negative BCC, MDA-MB-231 cells, (nEV) and those induced with the SARS-CoV-2 M-protein (MpEV) by ultracentrifugation. The size of nEV and MpEV were measured by dynamic light scattering and the expression of markers were examined by Western Blotting. Both nEV and MpEV were nanosized, ranging from 60 to 500 nm (**Supplementary Figure 1A**) and showed the expression of CD63, TSG101, and the negative expression of APOA1 (**Supplementary Figure 1B**).

**Figure 1:**
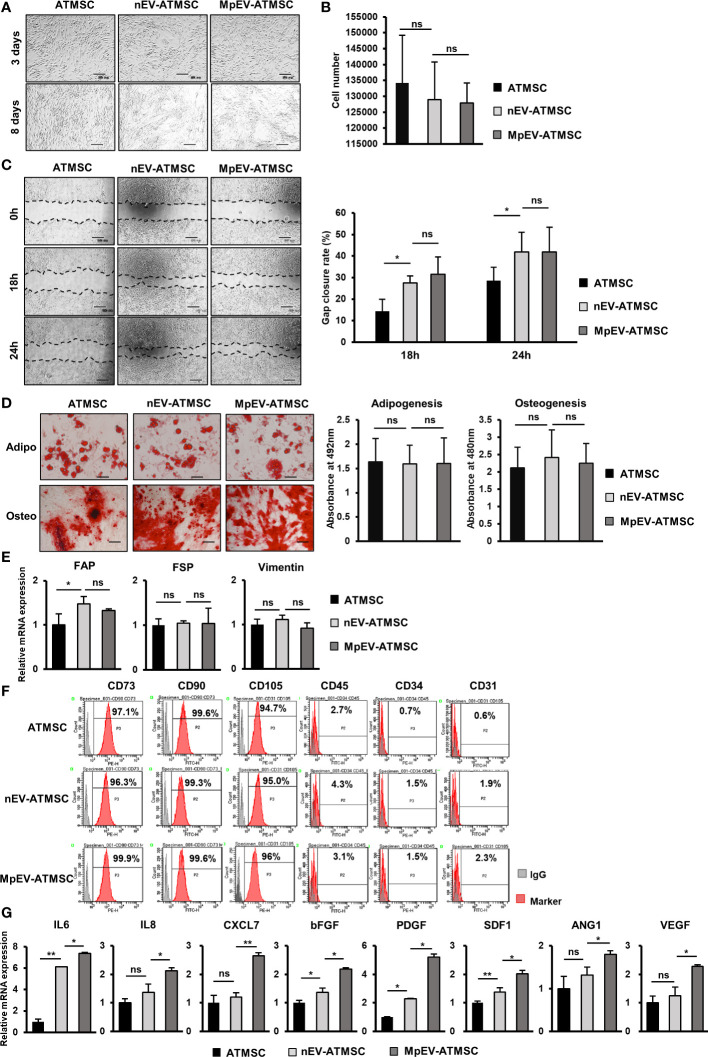
Effects of MpEV secreted from M-protein-induced triple negative BCC on ATMSC. **(A)** Morphology of ATMSC, nEV-ATMSC, MpEV-ATMSC (The scale bars indicate 500 µm). **(B)** Proliferation of ATMSC, nEV-ATMSC, MpEV-ATMSC. **(C)** Migration of ATMSC, nEV-ATMSC, MpEV-ATMSC (The scale bars indicate 500 µm). **(D)** Adipocyte and osteocyte differentiation of ATMSC, nEV-ATMSC, MpEV-ATMSC (The scale bars indicate 200 µm). **(E)** The cancer-associated fibroblasts marker expression of ATMSC, nEV-ATMSC, MpEV-ATMSC. **(F)** Characterized MSC markers of ATMSC, nEV-ATMSC, MpEV-ATMSC. **(G)** The cancer supporting gene expression of ATMSC, nEV-ATMSC, MpEV-ATMSC. The value represents the mean ± SD of triplicate experiments. (ns, no significance; p > 0.05; *p ≤ 0.05; **p ≤ 0.01).

Next, ATMSC were incubated with PKH26-labeled nEV or MpEV to mediate the uptake. The percentage of ATMSC uptaking of PKH26-labeled nEV or MpEV was 90%, examined by flow cytometry (**Supplementary Figure 1C**). Characterization of ATMSC post-uptaking EV showed that the uptake of either nEV or MpEV to ATMSC did not affect their morphology (**Figure 1A**) and proliferation (**Figure 1B**). Meanwhile, in comparison to the original ATMSC, those uptaking nEV or MpEV showed the induced migratory ability (**Figure 1C**). However, no different effects between nEV and MpEV on the migratory ability of ATMSC were observed (**Figure 1C**). In addition, the effects of nEV or MpEV on the abilities of ATMSC to differentiate to adipocytes and osteoblasts were assessed. The formation of adipocytes and osteoblasts was examined by staining with Oil red O and Alizarin red, respectively. As a result, the uptake of either nEV or MpEV to ATMSC did not affect their ability to differentiate into osteoblasts and adipocytes (**Figure 1D**). Next, the effects of MpEV on the cancer-associated fibroblast differentiation of ATMSC were examined by analyzing the gene expression of cancer-associated fibroblast markers in these cells. ATMSC which uptook either nEV (nEV-ATMSC) or MpEV (MpEV-ATMSC) showed no significant changes in the expression of cancer-associated fibroblast markers (e.g., FAP, FSP, Vimentin) (**Figure 1E**). In addition, neither nEV nor MpEV affected the expression of MSC markers (**Figure 1F**).

In the breast TME, ATMSC support the progression of breast tumors via paracrine effects. The secretion of a spectrum of pro-tumorigenic factors from ATMSC promotes the growth and metastasis of cancer cells (37). Therefore, we next examined the effects of nEV and MpEV on the expression of growth factors and cytokines that support cancer. As shown in **Figure 1G**, in comparison to the original ATMSC and nEV-ATMSC, MpEV-ATMSC showed the significant upregulation of genes related to the invasion, metastasis, and drug resistance of breast cancer and angiogenesis, including IL6, IL8, bFGF, PDGF, SDF1, ANG1, VEGF, and CXCL7 (38–42).

Taken together, these results suggest that neither nEV nor MpEV altered the phenotypes of ATMSC, including their morphology, differentiation ability, and marker expression. However, MpEV-ATMSC showed the increased expression of cancer-related growth factors and cytokines, suggesting their ability to support tumor growth.

### MpEV-ATMSC induced the metastasis of non-aggressive BCC via paracrine effects

**Figure 2:**
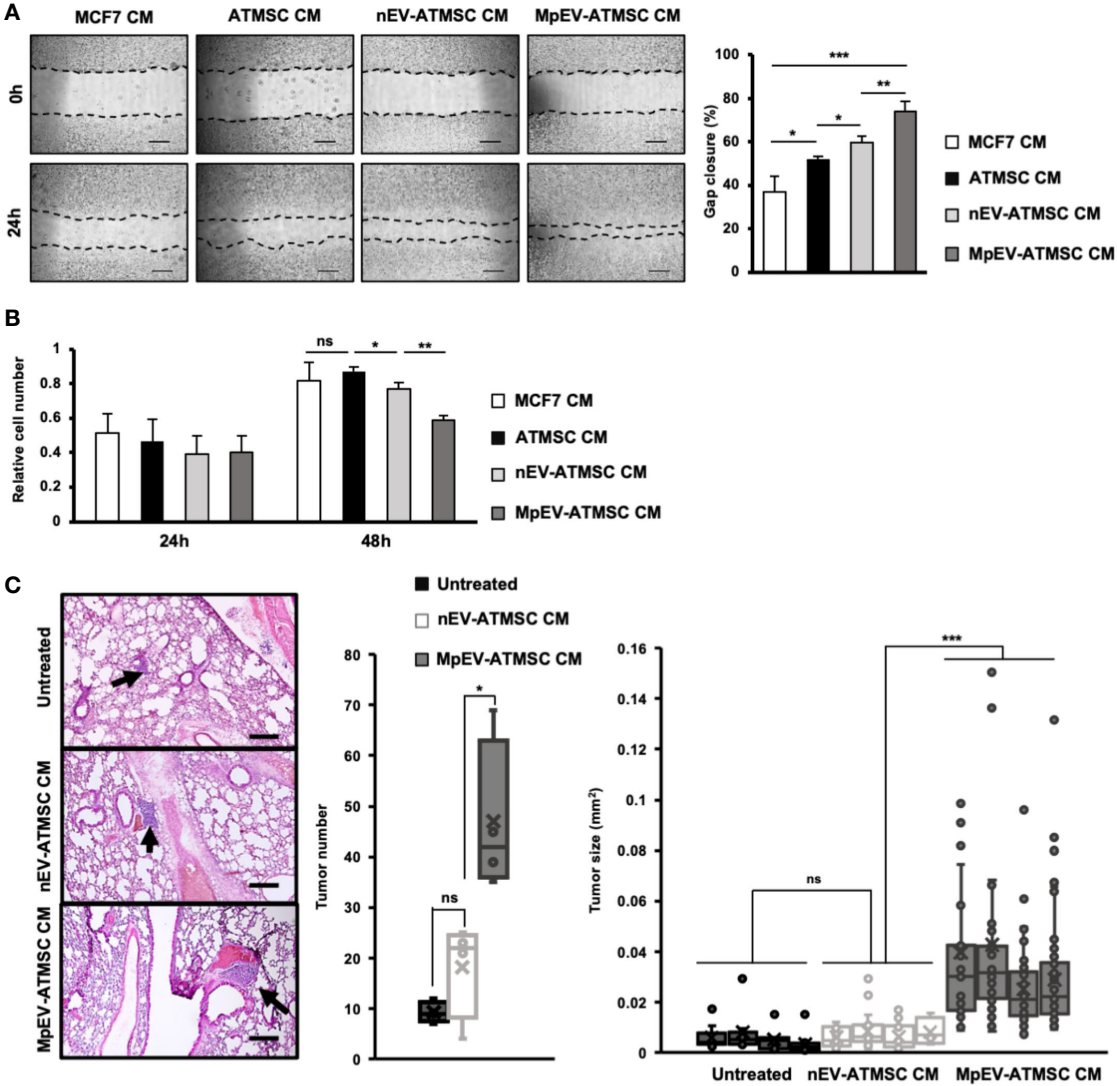
Paracrine effects of MpEV-ATMSC on BCC. **(A)** Migration of MCF7 cells treated with ATMSC, nEV-ATMSC, MpEV-ATMSC-derived CM (The scale bars indicate 500 µm). **(B)** Proliferation of MCF7 cells treated with ATMSC, nEV-ATMSC, MpEV-ATMSC-derived CM. **(C)** *In vivo* lung metastasis of MCF7 cells treated by CM derived from ATMSC, nEV-ATMSC, MpEV-ATMSC: HE staining of lung tissue (The arrows indicate tumors, the scale bars indicate 200 µm), number of tumor foci, and size of each tumor foci (each dot represents each tumor foci, each column represents each mouse). Each value represents the mean ± SD of triplicate experiments. (ns, no significance; p > 0.05; *p ≤ 0.05; **p ≤ 0.01; ***p ≤ 0.001).

We previously reported the induced metastasis of BCC treated with conditioned medium (CM) from ATMSC (25). Therefore, in order to examine the ability of MpEV-ATMSC to support breast cancer, CM from MpEV-ATMSC (MpEV-ATMSC-CM) was collected and used to culture non-aggressive BCC (MCF7). The results of a scratch assay showed that non-aggressive BCC treated with ATMSC-CM showed a significantly increased migratory ability in comparison to those cultured in normal medium (**Figure 2A**). Notably, non-aggressive BCC treated with MpEV-ATMSC-CM showed higher promotion of migration in comparison to those treated with CM derived from ATMSC or nEV-ATMSC (**Figure 2A**). Meanwhile, MpEV-ATMSC-CM reduced the proliferation of non-aggressive BCC after 48-hour treatment (**Figure 2B**) in comparison to those cultured under normal conditions. These data suggested that the higher percentage of gap closure in non-aggressive BCC treated with MpEV-ATMSC-CM was not due to the increased proliferation of these cells (**Figure 2A**). A similar tendency was observed in MDA-MB-231 triple-negative BCC, in which the migratory ability of MDA-MB-231 cells was also promoted by MpEV-ATMSC-CM, whereas their proliferation was reduced (**Supplementary Figures 2A, B**).

Next, the effects of MpEV-ATMSC-CM on *in vivo* metastasis of non-aggressive BCC were examined using a lung metastatic mouse model, in which mice were injected with non-aggressive BCC treated with CM from different ATMSC via tail vein. Mice injected with non-aggressive BCC treated with MpEV-ATMSC-CM showed a significantly higher number of tumor foci in the lungs in comparison to those injected with non-aggressive BCC treated with nEV-ATMSC-CM (**Figure 2C**). In addition, non-aggressive BCC treated with MpEV-ATMSC-CM showed the ability to form larger tumors in mouse lungs in comparison to those treated with nEV-ATMSC-CM (**Figure 2C**).

Taken together, these results suggested that MpEV significantly induced the ability of ATMSC to promote the migration of non-aggressive BCC *in vitro* and metastasis *in vivo*.

### PGE2/IL1 signaling pathway was involved in the induced tumorigenic ability of ATMSC by MpEV

**Figure 3:**
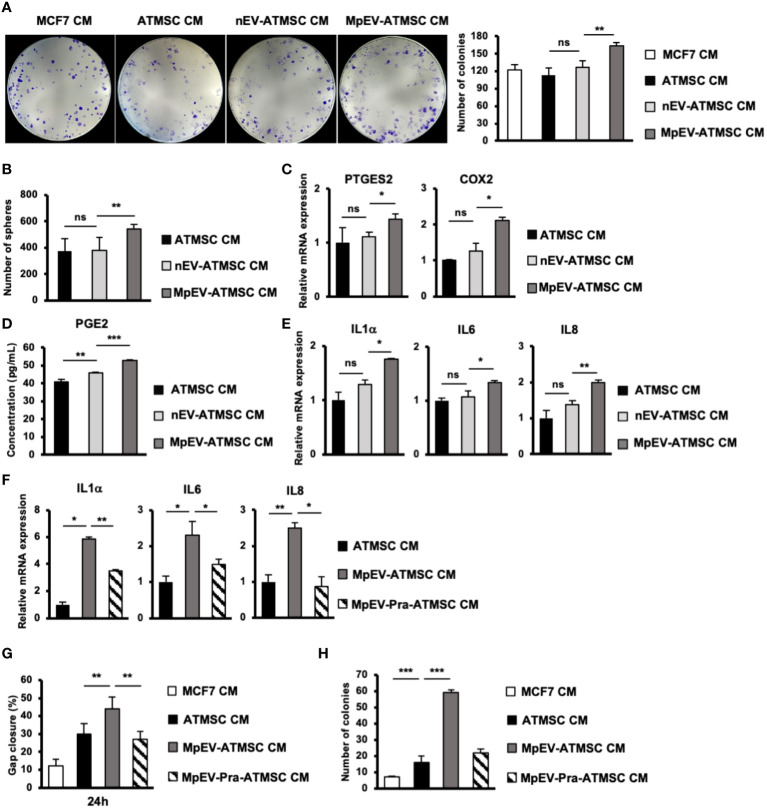
PGE2/IL1 signaling pathway was involved in the induced ability of MpEV-ATMSC to support cancer stemness. **(A)** Colony formation of MCF7 cells treated with CM derived from ATMSC, nEV-ATMSC, MpEV-ATMSC. **(B)** Mammosphere formation of MCF7 cells treated with CM derived from ATMSC, nEV-ATMSC, MpEV-ATMSC. **(C)** The expression of PTGES2 and COX2 in CM derived from ATMSC, nEV-ATMSC, MpEV-ATMSC. **(D)** Concentration of PGE2 in CM derived from ATMSC, nEV-ATMSC, MpEV-ATMSC measured by ELISA assay. **(E)** The expression of cytokines in MCF7 cells treated with CM derived from ATMSC, nEV-ATMSC, MpEV-ATMSC. **(F)** The expression of cytokines in MCF7 cells treated with CM derived from MpEV-ATMSC cultured in the presence of an inhibitor of PGE2 production. **(G)** Migration of MCF7 cells treated with CM derived from MpEV-ATMSC cultured in the presence of an inhibitor of PGE2 production. **(H)** Colony formation of MCF7 cells treated with CM derived from MpEV-ATMSC cultured in the presence of an inhibitor of PGE2 production. Each value represents the mean ± SD of triplicate experiments. (ns, no significance; p > 0.05; *p ≤ 0.05; **p ≤ 0.01; ***p ≤ 0.001).

Next, we examined the stemness potency of non-aggressive BCC in response to CM from ATMSC by colony formation and mammosphere formation assays. Sphere formation assay generates colony-forming units in 3D aggregates under a serum-free, nutritionally deficient and anchorage-independent culture conditions; therefore, cancer cells undergo apoptosis, while cancer stem cells still survive and proliferate. Meanwhile, colony formation assay examines the ability of single cells to initiate and growth into full colonies in very low density seeding in monolayer culture. The results showed that, in comparison to the original ATMSC-CM and nEV-ATMSC-CM, MpEV-ATMSC-CM significantly promoted colony formation (1.44 and 1.3 times, **Figure 3A**) and mammosphere formation (1.44 and 1.43 times, **Figure 3B**) of non-aggressive BCC, which implied their increased clonogenicity. The similar results, which the colony and mammosphere formation was induced in MDA-MB-231 triple-negative BCC-treated with MpEV-ATMSC-CM, were also observed (**Supplementary Figures 2C, D**).

Previous reports demonstrated that the interaction loop between MSC and cancer cells promoting cancer stemness is involved in the PGE2/IL1 signaling pathway (43). Therefore, we next examined the expression of PGE2 in MpEV-ATMSC. As shown in **Figure 3C**, in comparison to the control group without any treatment and the group treated with nEV, those treated with MpEV significantly increased the gene expression of PTGES2 and COX2, which are responsible for the production of PGE2 in ATMSC. Next the effects of MpEV on the secretion of PGE2 from ATMSC was examined by using an ELISA kit to measure the concentration of PGE2 in EV-free conditioned medium derived from ATMSC treated with MpEV. As a result, MpEV-ATMSC showed a higher secretion of PGE2, in comparison to nEV-ATMSC and the control ATMSC without any EV treatment (PGE2 concentration, Control ATMSC without any treatment: 40.85 ± 1.46 pg/mL, nEV-ATMSC: 46 ± 0.42 pg/mL, MpEV-ATMSC: 53.08 ± 0.05 pg/mL, **Figure 3D**).

PGE2 production was reported to be induced in ATMSC following their interaction with IL1 signaling in cancer cells. The secreted PGE2 and cytokines amplify the expression of cytokines (e.g., IL1, IL6, and IL8) in cancer cells, thereby activating their stemness (43). Therefore, we examined the expression of these cytokines in BCC. Consistent with the upregulation of PGE2 in MpEV-ATMSC, non-aggressive BCC treated with MpEV-ATMSC-CM showed the upregulation of IL1α, IL6, and IL8 in response to the stimulation signal from PGE2 in comparison to those treated with ATMSC-CM or nEV-ATMSC-CM (**Figure 3E**). This interaction was confirmed using the PGE2 inhibitor, pranoprofen. Non-aggressive BCC induced by CM from Pranoprofen-treated MpEV-ATMSC showed significantly reduced IL1, IL6, IL8 cytokine expression levels (**Figure 3F**), migratory ability (**Figure 3G**), and colony formation (**Figure 3H**), in comparison to non-aggressive BCC induced by CM from MpEV-ATMSC.

Taken together, these data suggest the role of the PGE2/IL1 signaling pathway in the interaction between MpEV-ATMSC and BCC to support the stemness of BCC.

### MpEV altered the characteristics of EPC

**Figure 4:**
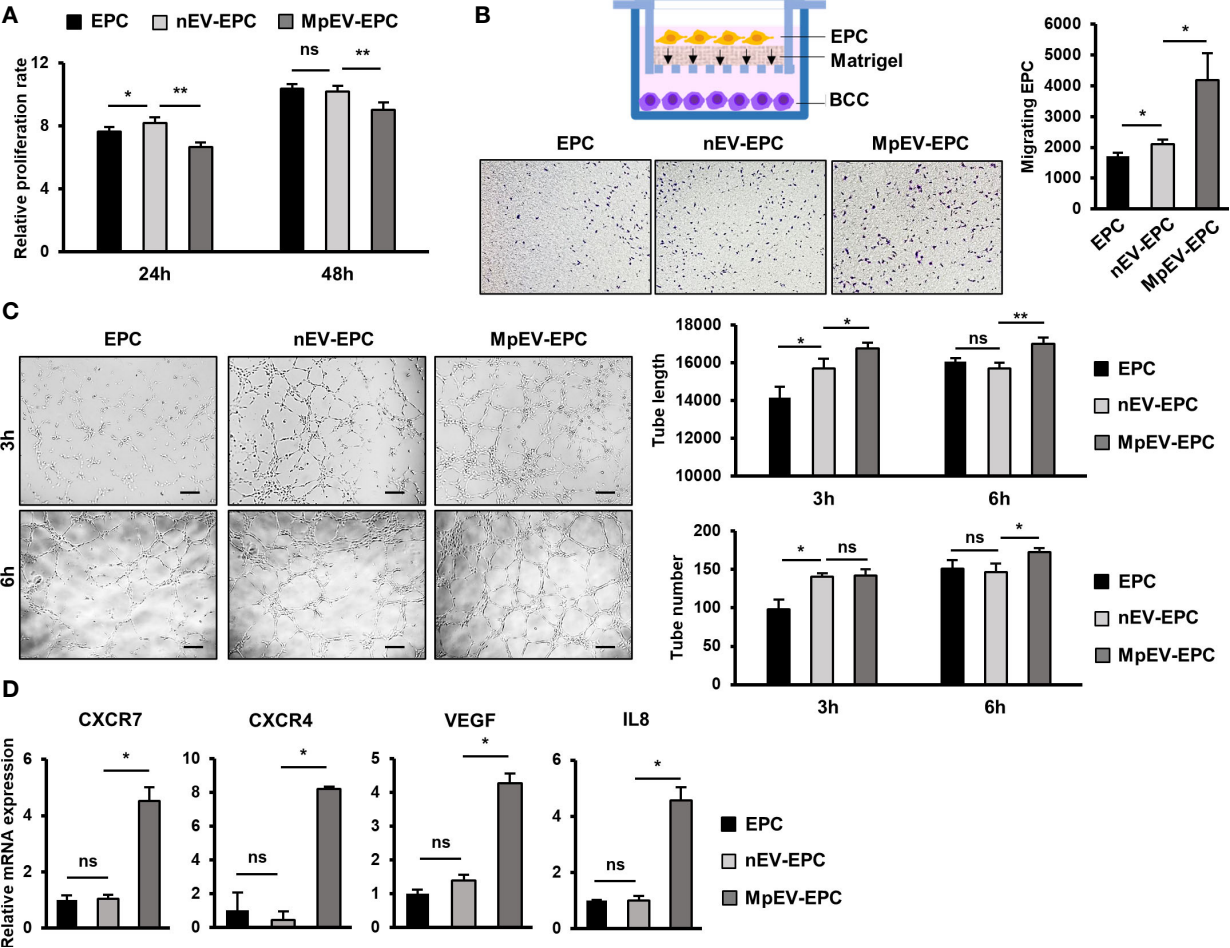
Effects of MpEV secreted from M-protein-induced triple negative BCC on EPC. **(A)** Proliferation of EPC, nEV-EPC, MpEV-EPC. **(B)** Transwell migration of EPC, nEV-EPC, MpEV-EPC. **(C)** Tube formation of EPC, nEV-EPC, MpEV-EPC. **(D)** The angiogenesis-related gene expression of EPC, nEV-EPC, MpEV-EPC. The scale bars indicate 200 µm. Each value represents the mean ± SD of triplicate experiments. (ns, no significance; p > 0.05; *p ≤ 0.05; **p ≤ 0.01).

We next examined whether MpEV show any effects on another type of tissue stem cells related to TME, in addition to ATMSC. In the breast TME, besides ATMSC, EPC is another type of tissue stem cells possessing the ability to differentiate into TEC, form new blood vessels, and secrete signals to promote cancer cells, thereby contributing to tumor growth (4). Therefore, we next incubated EPC with PKH-labeled nEV or MpEV to mediate the uptake of EV and examined the effects of MpEV on the functions of EPC in tumor development. The EV uptake by EPC was confirmed by a flow cytometry which showed that over 90% of EPC uptaking either nEV or MpEV (**Supplementary Figure 3A**). Next, the effects of MpEV on the proliferation of EPC were examined which showed that EPC uptaking MpEV (MpEV-EPC) exhibited a slightly reduced proliferation in comparison to the original EPC or those uptaking nEV (nEV-EPC) (**Figure 4A**). In addition, the results of the transwell migration assay showed a significantly increased migratory ability toward BCC in MpEV-EPC and nEV-EPC, in comparison to the original EPC (**Figure 4B**). Notably, MpEV-EPC showed a higher migratory ability toward BCC than nEV-EPC (**Figure 4B**).

Next, the effects of MpEV on the angiogenic ability of EPC were examined using a tube formation assay. EPC was seeded in the Matrigel-coated wells of a 24-well plate and the formation of tubes was observed and quantification after three and six hours. Intriguingly, the results showed that after 6 hours MpEV-EPC had an increased ability to form tubes, suggesting an increased angiogenetic ability, in comparison to nEV-EPC and the original EPC (1.18 and 1.07 times, respectively; **Figure 4C**). Moreover, in MpEV-EPC, the expression of genes related to angiogenesis (e.g., CXCR7, CXCR4, VEGF and IL8) was significantly induced in comparison to nEV-EPC and the original EPC (**Figure 4D**).

In addition, the effects of MpEV on endothelial cells (EC), the differentiated cells from EPC, were examined. After incubating with either nEV or MpEV and confirming the uptake of EV by EC (**Supplementary Figure 3B**), the migration, proliferation and tube formation ability of these EC were examined. Similar to the effects on EPC, MpEV promoted EC migration toward BCC signals in comparison to nEV (**Supplementary Figure 3C**). However, in contrast to the effects on EPC, MpEV promoted proliferation (**Supplementary Figure 3C**) and showed no effects on the angiogenic ability (**Supplementary Figure 3D**) of EC, in comparison to nEV. These results suggest different responses of stem cells and differentiated cells to MpEV signaling.

Taken together, these results suggest that the ability of EPC to migrate toward tumors and differentiate and incorporate themselves into newly formed blood vessels was strengthened by the induction of EV secreted from M-protein-induced triple-negative BCC.

### MpEV-EPC acquired TEC-like characteristics

**Figure 5:**
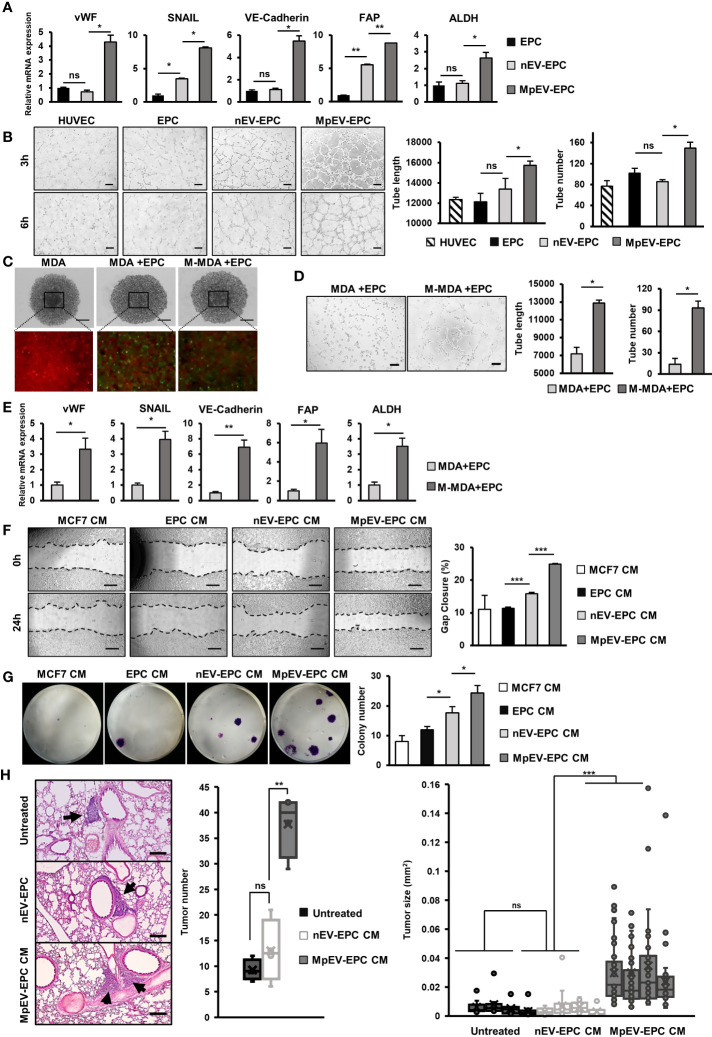
MpEV-EPC acquired TEC-like characteristics to support breast cancer cell progression. **(A)** The TEC-related gene expression of EPC, nEV-EPC, MpEV-EPC. **(B)** Tube formation in no-serum condition of EPC, nEV-EPC, MpEV-EPC (the scale bars indicate 200 µm). **(C)** 5-day spheroid culture of PKH26-labeled MDA-MB-231 cells and CFSE-labeled EPC (the scale bars indicate 500 µm, MDA: MDA-MB-231, M-MDA: M-protein-induced MDA-MB-231, Green: CFSE, Red: PHK26). **(D)** Tube formation of EPC isolated from spheroid (the scale bars indicate 200 µm, MDA: MDA-MB-231, M-MDA: M-protein-induced MDA-MB-231). **(E)** TEC-related gene expression of EPC isolated from spheroid (MDA: MDA-MB-231, M-MDA: M-protein-induced MDA-MB-231). **(F)** Migration of MCF7 treated with CM derived from EPC, nEV-EPC, MpEV-EPC (the scale bars indicate 500 µm). **(G)** Colony formation of MCF7 treated with CM derived from EPC, nEV-EPC, MpEV-EPC. **(H)** *In vivo* lung metastasis of MCF7 cells treated by CM derived from EPC, nEV-EPC, MpEV-EPC: HE staining of lung tissue (The arrows indicate tumors, the scale bars indicate 200 µm), number of tumor foci, and size of each tumor foci (each dot represents each tumor foci, each column represents each mouse). Each value represents the mean ± SD of triplicate experiments. (ns, no significance; p > 0.05; *p ≤ 0.05; **p ≤ 0.01; ***p ≤ 0.001).

Among non-cancer cells in the TME, TEC—which originate from EC or EPC—show the notable capability of supporting cancer cells (8). Therefore, we next examined the effects of MpEV on the ability of EPC to acquire TEC-like phenotypes by checking the expression of TEC markers in MpEV-EPC. The results showed that MpEV-EPC exhibited the significantly upregulated expression of vWF, SNAIL, VE-Cadherin, FAP, and ALDH (TEC markers found in breast tumors) (44–48) in comparison to nEV-EPC or the original EPC (**Figure 5A**). Next, the ability to form tubes without serum, a specific feature of TEC, was examined in MpEV-EPC. EPC was seeded in medium free serum in a Matrigel-coated well plate to induce the forming of capillary like structures (49). As shown in **Figure 5B**, while the control groups, including normal EC and EPC, hardly formed tubes, the uptake of nEV or MpEV induced tube formation in EPC under serum-depleted conditions. Notably, MpEV induced EPC tube formation in comparison to nEV (1.18 times, **Figure 5B**).

Next, to examine the characteristics of EPC under the effects of M-protein or MpEV in tumor microenvironment, we performed an *in vitro* 3D spheroid model which mimic the tumor microenvironment, reported by a recent study (50). Briefly, spheroids were developed by coculturing of PKH26-labeled BCC and CFSE-labeled EPC under the presence of M-protein for 5 days (**Figure 5C**). After that, the CFSE-labeled EPC was isolated by cell sorting, then the expression of TEC markers and the angiogenic ability in serum-free conditions of these EPC were accessed. The results showed that EPC isolated from spheroids with M-protein-treatment showed the induced gene expression of TEC markers (**Figure 5D**) and the promoted angiogenic ability under serum-depleted conditions (**Figure 5E**).

Next, the ability of MpEV-EPC to support non-aggressive BCC was examined by treating BCC with CM derived from EPC. As a result, non-aggressive BCC treated with CM derived from MpEV-EPC (MpEV-EPC-CM) showed significantly enhanced migratory ability (2.27-, 2.19 and 1.56 times, **Figure 5E**) and colony formation (3.04-, 2.03 and 1.38 times, **Figure 5F**), in comparison to the original non-aggressive BCC or non-aggressive BCC treated with CM derived from EPC or nEV-EPC. Furthermore, the effects of MpEV-EPC-CM on the *in vivo* metastasis of non-aggressive BCC were examined in lung metastatic mouse model. The results showed that non-aggressive BCC treated with MpEV-EPC-CM showed significantly enhanced metastatic ability to the lungs of mice in comparison to the original non-aggressive BCC or those treated with CM derived from nEV-EPC, as demonstrated by the increased number and size of tumor foci in the mouse lung tissues (**Figure 5G**).

In addition, the effects of MpEV-EPC-CM on MDA-MB-231 triple-negative BCC line were examined as a type of aggressive BCC. The results differed from the impact on non-aggressive BCC, in which MpEV-EPC-CM stimulated migration (**Supplementary Figure 4A**) but showed no effects on the proliferation (**Supplementary Figure 4B**) and colony formation (**Supplementary Figure 4C**) of MDA-MB-231 cells.

Taken together, EV derived from M-protein-induced triple-negative BCC altered the phenotypes of EPC, which acquired TEC-like characteristics, and induced the ability of EPC to promote metastasis of non-aggressive BCC.

### MpEV-ATMSC supported the angiogenesis of EPC

**Figure 6:**
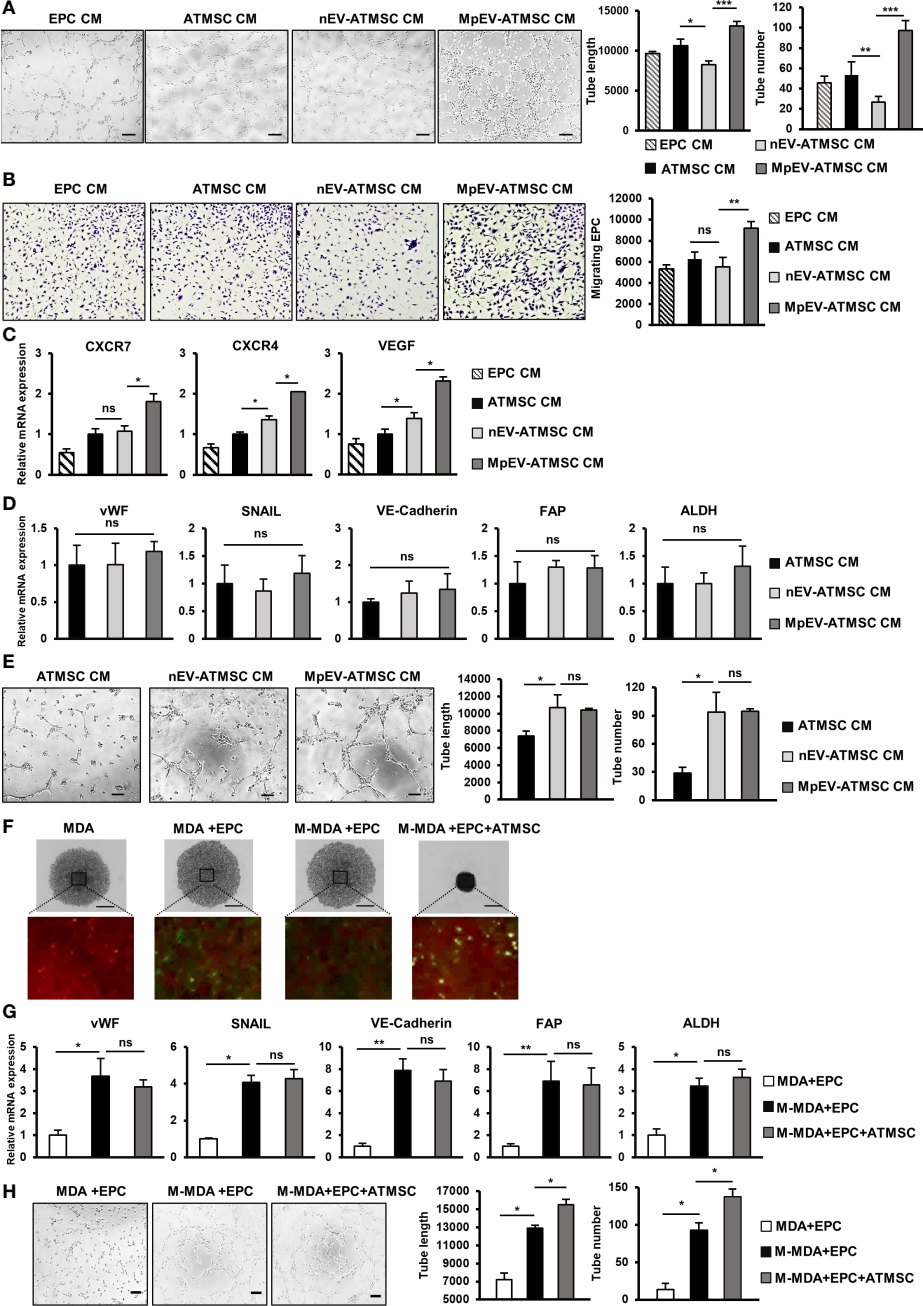
MpEV-ATMSC supported angiogenesis of EPC. **(A)** Tube formation of EPC treated by CM derived from ATMSC, nEV-ATMSC, MpEV-ATMSC (The scale bars indicate 200 µm). **(B)** Transwell migration of EPC treated by CM derived from ATMSC, nEV-ATMSC, MpEV-ATMSC. **(C)** Angiogenesis-related gene expression of EPC treated by CM derived from ATMSC, nEV-ATMSC, MpEV-ATMSC. **(D)** TEC-related gene expression of EPC treated by CM derived from ATMSC, nEV-ATMSC, MpEV-ATMSC. **(E)** Tube formation of EPC treated by CM derived from ATMSC, nEV-ATMSC, MpEV-ATMSC (The scale bars indicate 200 µm). **(F)** 5-day spheroid culture of PKH26-labeled MDA-MB-231 cells, CFSE-labeled EPC and ATMSC (the scale bars indicate 500 µm, MDA: MDA-MB-231, M-MDA: M-protein-induced MDA-MB-231, Green: CFSE, Red: PHK26). **(G)** TEC-related gene expression of EPC isolated from spheroid (MDA: MDA-MB-231, M-MDA: M-protein-induced MDA-MB-231). **(H)** Tube formation of EPC isolated from spheroid (The scale bars indicate 200 µm, MDA: MDA-MB-231, M-MDA: M-protein-induced MDA-MB-231). Each value represents the mean ± SD of triplicate experiments. (ns, no significance; p > 0.05; *p ≤ 0.05; **p ≤ 0.01; ***p ≤ 0.001).

In addition to the ability to support cancer cells, ATMSC also have the ability to support the functions of EPC and TEC in a paracrine manner via their secreted factors (51–53). As MpEV-ATMSC showed the upregulation of angiogenic factors, such as bFGF, SDF1, PDGF, ANG1, VEGF, and CXCL7 (**Figure 1G**), we next investigated the ability of MpEV-ATMSC to support the angiogenic ability of EPC by a tube formation assay. EPC were treated with CM derived from MpEV-ATMSC (MpEV-ATMSC-CM) and their angiogenic abilities were examined by a tube formation assay using a Matrigel-coated 24-well plate. The results showed that EPC treated with MpEV-ATMSC-CM exhibited significantly increased tube formation ability in comparison to those treated with nEV-ATMSC-CM (1.59-fold increased, **Figure 6A**).

In addition, the effects of MpEV-ATMSC-CM on the migration of EPC toward signals from BCC were examined using a transwell insert system. Treatment with MpEV-ATMSC-CM significantly promoted the migration of EPC toward BCC, while ATMSC-CM and nEV-ATMSC-CM showed no effect (**Figure 6B**). Consistent with the induced angiogenic and migratory abilities, EPC treated with MpEV-ATMSC-CM showed a significant upregulation of genes related to angiogenesis and migration, such as CXCR7, CXCR4, and VEGF, in comparison to the original EPC and those treated with ATMSC-CM or nEV-ATMSC-CM (**Figure 6C**). Moreover, the effects of MpEV-ATMSC-CM on the gene expression of TEC markers and the tube formation of EPC under free serum condition were examined. The results showed that treatment with MpEV-ATMSC-CM showed no significant effects to induce the gene expression of TEC markers in EPC (**Figure 6D**) and the tube formation ability of EPC in serum-depleted condition, in comparison to nEV-ATMSC-CM (**Figure 6E**).

Moreover, to examine the contribution of ATMSC in the signaling network of BCC and EPC under the effects of M-protein, an *in vitro* 3D spheroid triculture of PKH26-labeled BCC, ATMSC and CFSE-labeled EPC was performed (**Supplementary Figure 5**). After 5 days, spheroids formed in the presence of ATMSC showed an increased condensation with smaller sizes than those without ATMSC, suggesting that ATMSC might promote the aggregation of cells inside the spheroids (**Figure 6F**). Next, the CFSE-labeled EPC was isolated from spheroids by cell sorting and the characteristics of TEC were accessed. As a result, compared to EPC isolated from spheroids without M-protein treatment, those isolated from spheroids with M-protein-treatment showed the induced gene expression of TEC markers (**Figure 6G**) and tube formation ability under serum-depleted conditions (**Figure 6H**). Notably, among the M-protein-treated groups, EPC isolated from spheroids with the presence of ATMSC showed the comparable gene expression of TEC markers (**Figure 6G**), but higher tube formation ability under serum-depleted condition, in comparison to those isolated from spheroids without ATMSC (**Figure 6H**). These data suggested that ATMSC did not change the phenotypes of EPC to TEC but supported the tube formation ability of EPC under a tumor microenvironment mimic condition.

Taken together, these results suggest that the uptake of MpEV induces the paracrine effects of ATMSC, supporting the migration of EPC toward tumor sites and the angiogenic ability of EPC.

## Discussion

**Figure 7:**
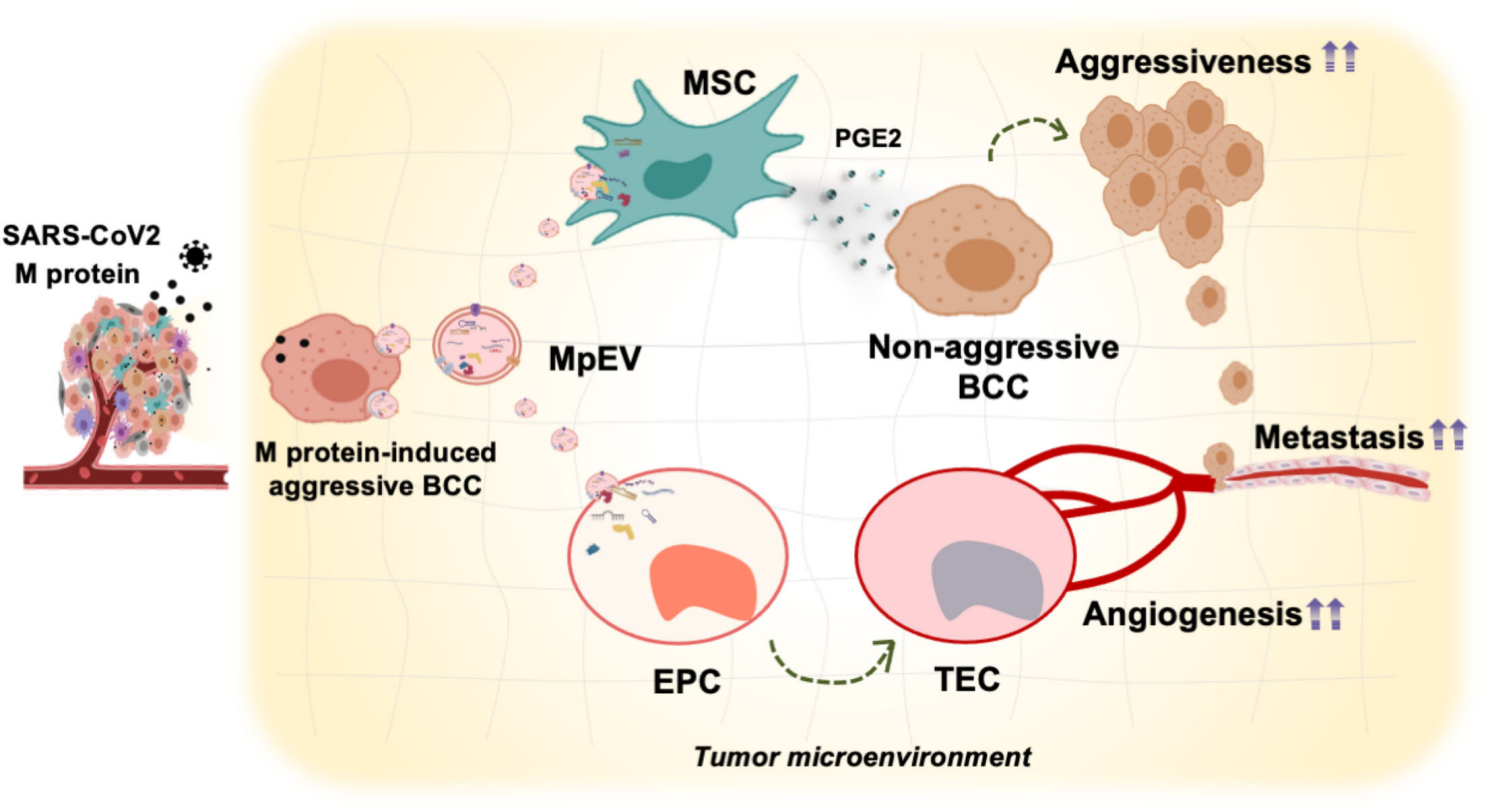
Proposed model: In TME, SARS-CoV-2 M-protein-induced BCC recruit and control the activity of ATMSC and EPC via EV which facilitate the development and metastasis of breast tumors.

Our present study demonstrated that M-protein induced the ability of triple-negative BCC-derived EV promoting the functions of breast tissue stem cells including ATMSC and EPC in tumorigenesis. ATMSC uptaking of EV derived from M-protein-induced triple-negative BCC (MpEV) showed the induced paracrine effects on the malignancy of non-aggressive BCC, including migration, metastasis, and stemness potency, which was involved in the increased secretion of PGE2. Meanwhile, EPC uptaking of MpEV acquired tumor endothelial cell-like phenotype, with promoted abilities in angiogenesis and facilitating the metastasis and stemness characteristics of non-aggressive BCC (**Figure 7**).

Numerous studies have suggested that COVID19 increases the risk of accelerated cancer progression, metastasis, and death in cancer patients in general and in breast cancer patients in particular (5, 12, 13, 54, 55). Indeed, our previous study reported the induced effects of the SARS-CoV-2 M-protein on the migratory ability and metastasis of BCC, suggesting that it promoted aggressiveness in BCC (6). Several studies have reported the effects of SARS-CoV-2 infection on the TME, mainly focusing on acute inflammation and immune reactions. In addition, cancer cells have also been observed to be affected by the virus. In lung cancer, the abundant appearance of whole SARS-CoV-2 proteins has been shown to disrupt the immune system, cause cytokine storms, and alter metabolism in the TME, resulting in increased tumor growth (56). Moreover, a report on breast cancer and melanoma showed alterations in the immune response of the TME by direct injection of inactivated SARS-CoV-2 (57, 58). However, to date, the effects of SARS-CoV-2 infection and its proteins on the interactions between cancer cells and other cells in the TME after SARS-CoV-2 infection have not been discussed.

To gain and maintain a suitable surrounding environment, cancer cells actively contact and control the activities of neighboring cells in numerous ways, including the secretion of extracellular vesicles (EV) (30). EV derived from cancer cells were uptaken by the surrounding cells and regulate their behaviors to support tumor development. Although the effects of SARS-CoV-2 proteins on cancer cells have been widely reported, whether these proteins also affect EV derived from cancer cells are still obscured. Based on our previous findings of the induced malignancy of triple negative BCC by SARS-CoV-2 membrane protein (M-protein) (6), we expanded the study to examined the effects of M-protein on EV derived from triple negative BCC. We firstly isolated EV from the original triple negative BCC (nEV) and those with M-protein treatment (MpEV), then compared their effects on the triple negative BCC which received no treatment of M-protein. The results showed that in comparison to nEV, MpEV showed no different effects on the migration of BCC in a scratch assay (**Supplementary Figure 6A**) and the colony formation of BCC (**Supplementary Figure 6B**). However, MpEV induced a higher proliferation (**Supplementary Figure 6C**), cytokine gene expression such as IL6, IL8 and TNFα (**Supplementary Figure 6D**) and HIF1α (**Supplementary Figure 6E**) in BCC, in comparison to nEV. These data suggested the different effects of MpEV on the recipient cells in comparison to nEV, which confirmed the hypothesis that M-protein also affects the functions of EV derived from BCC.

TME consists of numerous types of cells including cancer cells, tissue stem cells and the other non-cancerous cells. We previously reported the ability of ATMSC, a type of tissue stem cells, supporting the metastasis of triple negative BCC via a paracrine effect. Therefore, in this study, we questioned whether MpEV affects the abilities of ATMSC supporting BCC. Although previous studies have reported that human MSC lack ACE2 and TMPRSS2 expression and are resistant to SARS-CoV-2 infection (46, 47), in the present study, our data showed that the M-protein indirectly affected the gene expression of ATMSC via MpEV derived from M-protein-induced triple-negative BCC. The uptake of MpEV by ATMSC upregulated the expression of several genes involved in tumor development (**Figure 1G**). Consistently, nonaggressive BCC treated with conditioned medium derived from MpEV-ATMSC showed the induced migration (**Figure 2A**), *in vivo* metastasis (**Figure 2C**) and stemness potency (**Figures 3A, B**) while reduced the proliferation (**Figure 2B**). Although proliferation is necessary for the initiation of primary tumors, growth inhibition plays an important role for the survival of tumor cells in the circulation and invasion to secondary organs, resulting to a malignant phenotype (59, 60). Therefore, it is worthy for a further study to examine the correlation between the reduced proliferation and malignant phenotypes of non-aggressive BCC treated with conditioned medium from MpEV-ATMSC by clarifying the signaling pathways regulating proliferation of these cells.

Notably, MpEV-ATMSC showed the upregulation of PTGES2 and COX2 (**Figure 3C**), two factors responsible for PGE2 production, and the secretion of PGE2 (**Figure 3D**) which are known to be involved in the interaction between TA-MSC and cancer cells (59). A previous study reported that the PGE2/IL1 cytokine network mediates the interaction between TA-MSC and colon cancer cells, in which PGE2 and cytokines derived from cancer-educated MSC enhance IL1, IL6, and IL8 production in cancer cells, which in turn enriches the cancer stem cell population (43). In our study, the treatment of non-aggressive BCC with CM derived from MpEV-ATMSC induced the expression of IL6, IL8, and IL1α in these BCC (**Figures 3E, F**), suggesting the involvement of the PGE2 signaling pathway in the ability of MpEV-ATMSC to induce cancer stemness.

MSC have flexible fate determination controlled by cancer cells (26, 61). Previous studies reported that BCC recruits MSC to the TME and educates them to transform them into MSC (TA-MSC) or cancer-associated fibroblasts (CAF) (62–66). In our study, MpEV-ATMSC showed no upregulation of CAF markers, while expressed MSC typical markers and differentiation ability to adipocytes and fibroblast (**Figures 1D, F**). These data suggested that MpEV-ATMSC retained the phenotypes of MSC but exhibited a higher ability to induce tumorigenesis in non-aggressive BCC, in comparison to the original ATMSC or those internalized with nEV. Meanwhile, in comparison to naïve MSC, TA-MSC possess a higher ability to promote the development of tumors; for instance, a study reported that gastric TA-MSC co-injected with gastric cancer cells highly increased tumor growth (67) in comparison to non-cancerous MSC such as bone marrow MSC or MSC derived from adjacent tissues (68). In addition, previous studies have reported that TA-MSC secreted bFGF, PDGF, and SDF1 to recruit more naive MSC into the TME (69, 70); and IL6 to enhance tumor cell growth (71). In our study, MpEV-ATMSC showed the upregulation of IL6, bFGF, PDGF, and SDF1 (**Figure 1G**) and the induced abilities to promote the malignancy of BCC, suggesting that MpEV-ATMSC might acquire TA-MSC-like characteristics.

In addition to ATMSC, we also examined the effects of MpEV on the behaviors of EPC, another type of tissue stem cell involved in tumor tissues and angiogenesis (30, 72, 73). Angiogenesis of tumors can be initiated when EPC are recruited to the TME by signaling factors from cancer cells and educated to differentiate into TEC and form new blood vessels, which ultimately allow a better supply of nutrients to the tumor and promote its development (33). In our study, after uptake of MpEV, angiogenic ability and migration toward BCC was induced in EPC (**Figures 4B, C**). In contrast, MpEV did not affect the angiogenic ability of EC (**Supplementary Figure 3C**). These results suggest that the flexibility of stem cells to be educated by cancer cells is greater than that of their mature counterparts. In comparison to normal EC, TEC show the higher expression of several specific genes that are considered to be tumor endothelial markers (74). Interestingly, our data showed that MpEV-EPC showed the upregulation of vWF, SNAIL, VE-Cadherin, FAP, and ALDH (**Figure 5A**), which are tumor endothelial markers associated with breast TEC (44–48). In addition, our data in **Figure 5B** show that MpEV-EPC exhibited breast TEC-like behavior with increased tube formation ability *in vitro* in the absence of serum, as reported in a previous study (47, 75). Notably, MpEV-EPC significantly induced aggressive phenotypes and *in vivo* metastasis of BCC (**Figures 5F–H**), which correlated with the increased expression of IL8, CXCR7, CXCR4, and VEGF (**Figure 4D**). IL8 from TEC was reported to play a mediating role in prostate cancer progression (76); meanwhile, CXCR7, CXCR4, and VEGF are angiocrine factors TEC stimulate angiogenesis and enhance survival of TEC in an autocrine manner (35, 77), thus supporting tumor progression and metastasis (78). Moreover, chemokine receptors CXCR7 and CXCR4 from TEC facilitate lymphoma and BCC transendothelial migration under the control of TEC (79).

In addition to signals from cancer cells, EPC and their angiogenesis in the TME are also affected by TA-MSC. TA-MSC in the TME have the ability to direct tumor angiogenesis by secreting angiogenic factors (26, 28, 80). In breast cancer, TA-MSC recruit EPC by secreting SDF1 and promoting the growth of tumor blood vessels (81). In our study, MpEV-ATMSC showed upregulation of growth factors and cytokines, including bFGF, PDGF, SDF1, and IL6 (**Figure 1G**), all of which contribute to the recruitment of EPC and driving angiogenesis (26, 40, 65). Consistently, conditioned medium derived from MpEV-ATMSC promoted tube formation, cancer-directed migration ability, and the expression of angiogenic genes in EPC, but showed no effects on the altered phenotypes of EPC to TEC-like cells (**Figures 6A–E**).

Of note, the contribution of ATMSC to the signaling network of M-protein-induced BCC and EPC was examined by a 3D spheroid triculture. After 5 days of triculture with M-protein-induced BCC, although EPC isolated from spheroids containing ATMSC showed no upregulation of TEC marker expression (**Figure 6G**), these cells exhibited the higher tube formation ability in serum free conditions (**Figure 6H**), in comparison to EPC isolated from spheroids without ATMSC. To directly examine the effects of MpEV under *in vitro* TME-mimic conditions, the spheroid triculture of BCC, EPC and ATMSC was performed with the addition of MpEV (**Supplementary Figure 7A**). Similarly, the results EPC isolated from spheroids with MpEV treatment showed the upregulation of TEC marker gene expression (**Supplementary Figure 7B**) and tube formation ability in serum free conditions (**Supplementary Figure 7C**), in comparison to those isolated from spheroids without MpEV treatment. Of note, the presence of ATMSC in the spheroid triculture did not induce the gene expression of TEC markers but promoted the tube formation ability of EPC in serum free conditions (**Supplementary Figure 7**). These data suggested that although showed no effects to alter EPC phenotype, ATMSC might contribute to promoting the angiogenic ability of EPC in the TME. In addition, MpEV induced the paracrine effects of ATMSC in both BCC and other non-cancer cells that are involved in facilitating the TME which is worthy for a further *in vivo* study.

In the TME, aside of tissue stem cells, cancer cells are supported by the other non-cancerous neighboring cells, such as cancer-associated fibroblasts (CAFs) and immune cells that supply nutrients, growth factors, cytokine signals, and blood vessel systems (26). In addition, a previous study showed that cancer cells with high malignancy tend to manipulate the surrounding cells more efficiently (27). Therefore, it is implied that BCC induced by the SARS-CoV-2 M-protein might possess a greater capability to regulate their TME. Indeed, our present study showed that MpEV derived from BCC induced by M-protein promoted the abilities of ATMSC and EPC to support cancer metastasis and malignancy. Therefore, it is worthy for a further study to examine the effects of MpEV on the other cell types in the TME such as CAFs and immune cells.

## Conclusion

In this study, we examined how SARS-CoV-2 M-protein modulates the tissue stem cells involved in breast TME by altering the functions of EV derived from triple-negative BCC. Our findings suggest that triple-negative BCC induced by M-protein produced EV with a higher ability to promote the functions of tissue stem cells, such as ATMSC and EPC, supporting cancer growth and aggressiveness (**Figure 7**). Our study suggests that SARS-CoV-2 infection in breast cancer patients not only promotes the aggressiveness of BCC themselves, but also the ability of BCC to manipulate the surrounding TME. By understanding the specific interactions between BCC and non-cancer cells, as well as the underlying mechanisms governing these interactions that occur during COVID-19, appropriate management of both medical conditions can be addressed, as well as identifying possible ways to prevent the exacerbation of breast cancer cells and their metastasis. Targeting the TME, especially tissue stem cells such as ATMSC, EPC, and their derivatives, should be considered as cancer therapeutic agents for cancer patients with COVID-19 infection.

## Materials and methods

### Breast cancer cell culture and induction with SARS-CoV-2 M-protein

MDA-MB-231 (ATCC HTB-26) cells (a triple-negative BCC line) and MCF-7 cells (a Luminal A BCC line [ATCC HTB-22]) were cultured in a culture dish containing Iscove’s modified Dulbecco’s medium (IMDM) (Gibco, Waltham, MA, USA) with 1% penicillin/streptomycin (Thermo Fisher Scientific, Waltham, MA, USA) and 5% fetal bovine serum (FBS) (Sigma-Aldrich, St. Louis, MO, USA). The cell medium was changed every two days and the cells were kept in a humidified incubator at 37°C under 5% CO_2_. The cells were subcultured to obtain 3.8×10^4^ cells/ml of medium per dish via trypsinization upon reaching 80% confluence.

MDA-MB-231 cells (5×10^5^ cells/ml) were treated with 60 pmol/ml SARS-CoV-2 M-protein (Miltenyi Biotec, Cologne, Germany) in EV-depleted FBS-containing medium for collecting EV in 5 days to collect CM and isolate EV. EV-depleted FBS-containing medium for collecting EV was prepared by ultracentrifugation using an Optima L-XP ultracentrifuge (Beckman Coulter, Inc. Brea, CA, United States), as previously described (82). Briefly, 40 ml of IMDM containing 5% FBS and 1% penicillin/streptomycin was ultracentrifuged at 140,000×*g* for 18 h at 4°C using a Beckman Coulter Type 70 Ti Rotor. Then, 30 ml of supernatant was collected and used as the culture medium to collect EV.

### Collection of EV

The CM of M-protein-treated and untreated BCC MDA-MB-231 cells was collected and centrifuged at 300×*g* for 5 min, followed by 1200×*g* for 20 min. The supernatant was ultracentrifuged at 140,000×*g* for 70 min at 4°C using an Optima XE-100 ultracentrifuge to collect the EV pellet. EV pellets were collected in 100 μL of PBS; these were considered to be isolated EV. The EV collected from the M-protein-treated BCC were called MpEV, and the EV collected from the untreated breast cancer cells were called nEV. For PKH26 labeling of EV, of 120 µg was resuspended in 250 µL of Diluent C (Sigma-Aldrich) and mixed with 250 µL of Diluent C containing 1 µL of PKH26 (Sigma-Aldrich). The mixture was then incubated in the dark for 5 min and neutralized by adding 40 ml of PBS containing 0.25% FBS. The solution was then ultracentrifuged at 140,000×*g* for 70 min at 4°C to collect stained EV pellet, then resuspended in 100 µL PBS and stored at -80°C. The protein concentration of EV was measured using a Bradford assay (Bio-Rad, Hercules, CA, USA) and was considered to be the EV concentration. The size of collected EV was measured by dynamic light scattering (Zetasizer Nano ZS, Melvern Instruments, United Kingdom). EV markers expression was characterized by Western Blotting.

### Culturing of ATMSC, EPC, and EC

ATMSC were isolated from human adipose tissues with permission from the ethics authorities at the University of Tsukuba [previously described by Kimura et al. (83)] and cultured in a culture dish containing IMDM with 10% FBS, 2 mg/ml L-glutamine (Invitrogen, Waltham, MA, US), 100 units/ml penicillin, and 5 ng/ml bFGF (Peprotech, Rocky Hill, NJ, USA).

EPC were isolated from umbilical cord blood [previously described (84)] and cultured in a dish containing IMDM with 10% FBS, 2 mg/ml L-glutamine (Invitrogen, Waltham, MA, US), 100 units/ml penicillin, and 5 ng/ml bFGF (Peprotech, Rocky Hill, NJ, USA).

For EC culture, human umbilical vein endothelial cells (HUVEC) were purchased from ATCC and cultured in a culture dish containing IMDM with 10% FBS, 100 units/ml penicillin/streptomycin, 0.2 µl/ml bFGF, and 0.5 µl/ml VEGF (Peprotech, Rocky Hill, NJ, USA). All cells were maintained in the incubator, the medium was changed every 2 days, and the cells were subcultured upon reaching 80% confluence, to obtain 3.8×10^4^ cells/ml of medium/dish.

### EV treatment for ATMSC, EPC and EC

ATMSC were seeded at a density of 1×10^5^ cells/ml in a 24-well culture plate, then treated with PKH26-labeled EV at an amount of 25 µg cultured for 4 days, followed by medium changing and another EV treatment for the next 4 days before examination. EPC or EC were seeded at a density of 1×10^5^ cells/ml in a 24-well culture plate and then treated with 10 µg or 5 µg PKH26-labeled EV, respectively (which ensured over 90% uptake, **Supplementary Figures 3A, B**) cultured for 2 days before examination. The uptake of PKH26-labeled EV to target cells was examined by a flow cytometer (BD LSRFortessa X-20; BD Biosciences).

### Collection of ATMSC- and EPC-derived CM

After EV treatment, ATMSC and EPC were washed with PBS before changing to a new corresponding medium without EV. After 48 h, the medium was collected and centrifuged at 300 g for 5 min, followed by 1200*xg* for 20 min (both at 4°C) to collect the supernatant. The collected CM was stored at -30°C.

### CM treatment of MDA-MB-231 and MCF7 BCC

MDA-MB-231 and MCF7 cells were seeded at a density of 1×10^5^ cells/ml in a 24-well culture plate for 2 h, and then the medium was replaced with ATMSC- or EPC-derived CM and cultured for 2 days before examination.

### Differentiation of ATMSC to adipocytes and osteoblasts

ATMSC was seeded at a number of 1×10^5^cells/well in a 4-well plate in MSC culture medium. After getting 100% confluency, the culture medium was changed to adipogenic or osteogenic differentiation medium. The culture medium was changed twice per week for 3 weeks. Adipogenic differentiation medium consisted of IMDM supplemented with 10% FBS, 0.1 mM dexamethasone (Sigma-Aldrich), 0.5 mM 3-isobutyl-1-methylxanthine (Sigma-Aldrich), 2 mg/mL insulin (Wako), and 0.1 mM indomethacine (Sigma-Aldrich). The formation of adipocytes was examined by staining with Oil Red O solution (Muto Pure Chemicals, Tokyo, Japan). For quantification, cells were dissolved with 4% IGPAL CA630 (Sigma-Aldrich) in isopropanol, and the absorbance at 492 nm was measured. Osteogenic differentiation medium consisted of IMDM supplemented with 1% FBS, 0.1 mM dexamethasone (Sigma-Aldrich), 10 mM β-glycerol-2-phosphate (Sigma-Aldrich), 0.2 mM ascorbic acid (Sigma-Aldrich), and 50 ng/mL human EGF (Wako). The formation of mineralized matrix was examined by staining with Alizarin Red S (Kodak, Rochester, NY). For quantification, cells were dissolved with 0.2 N HCl (Wako) and 5% sodium dodecyl sulfate, then the absorbance at 480 nm was measured.

### Analysis of MSC marker expression by flow cytometry

A number of 1×10^5^ ATMSC was collected in 200 μL PBS containing 2% FBS. After that, ATMSC were incubated with antibodies for 30 minutes at 4°C, including FITC-labeled anti-CD90 (BioLegend, San Diego, CA; 328107), PE-labeled anti-CD105 (BioLegend; 323206), PE-labeled anti-CD73 (BD Biosciences, San Diego, CA, 550257), FITC-labeled anti-CD31 (BioLegend; 303103), FITC–labeled anti-CD45 (BD Biosciences; 560976) and PE-labeled anti-CD34 (BD Biosciences; 560941). Cells stained with PE-labeled anti-IgG1 (555749; BD Biosciences), and FITC-labeled anti-IgG1 (555748; BD Biosciences) were used as the isotype controls. After that, cells were washed with PBS and resuspended in 300 μL PBS containing 2% FBS. The expression of MSC markers was analyzed by a flow cytometer (BD LSRFortessa X-20; BD Biosciences).

### ELISA for PGE2 quantification

CM from ATMSC was collected as described above. PGE2 concentration in CM was measured using ELISA kit Parameter Prostaglandin E2 assay (Biotechne R& D system, Mineapolis, USA). In short, CM and PGE2 standard with serial two-fold diluted concentration from 2500 pg/ml to 39 pg/ml were incubated in 96-well microplate together with primary antibody in 1 hour. The mixtures were then incubated with PGE2 conjugate in 2 hours and removed. After washing, the wells were incubated with substrate solution in 30 minutes, then reaction was stopped with acidic stop solution. Optical density at 450nm and 570nm (OD_450nm_ and OD_570nm_) was measured using microplate reader. OD value of each sample was calculated as follows:


$$\text{OD sample}=\text{ average }\left(\text{O}{\text{D}}_{450\text{nm sample}}-\text{O}{\text{D}}_{570\text{nm sample}}\right)-\text{ average }\left(\text{O}{\text{D}}_{450\text{nm blank}}-\text{O}{\text{D}}_{570\text{nm blank}}\right)$$

PGE2 concentration of each sample was calculated based on linear regression of OD value of standard curve in Microsoft Excel.

### Migration assay

ATMSC, MDA-MB-231, or MCF7 cells (2×10^5^ cells/400 µL/well) were seeded into 24-well plates in their respective culture medium and incubated for 24 h. Mitomycin C solution (Nacalai Tesque, Kyoto, Japan) was added at a concentration of 10 μg/ml and incubated for 1 h prior to creating a single scratch through the seeded cells using a 100 µl micropipette tip. The medium was removed and replaced with IMDM containing 0.25% FBS. Images of the scratch were taken immediately after the scratch and at 6-hour intervals up to 30 h or until closed using the Keyence BZ-XY710 microscope system (Keyence Corporation, Osaka, Japan). The gap closure percentage was analysis using ImageJ (NIH, Bethesda, MD).

### Proliferation assay

After treatment, ATMSC, EPC, HUVEC, MDA-MB-231 cells, and MCF7 cells were seeded into a 96-well plate to obtain 1×10^4^ cells/100 µl medium in each well. At 24 and 48 h, the absorbance at 450 nm (OD_450nm_) was measured after an hour of adding Cell Counting Kit-8 (Dojindo Molecular Technologies, Kumamoto, Japan).

### Transwell migration assay

MCF7 cells (5×10^4^ cells/600 µL of medium) were seeded into the wells of a 24-well plate for 48 h. After treatment, EPC or HUVEC (2×10^5^ cells/ml medium) were transferred into 8.0-µm pore cell transwell culture inserts (BD Falcon), which were then placed into the wells seeded with MCF7 cells. The cells were incubated for 6 h before staining. The cells were fixed with 4% paraformaldehyde before removing the cells on the membrane surface inside the transwells using a cotton swab. The remaining cells were permeabilized with methanol for 10 min, stained with a 2% crystal violet dye solution for 5 min, and then washed with distilled deionized water. Images of the transwell membrane were taken under a dissecting microscope, and the number of cells on the membrane was counted in 39 random areas on a created grid to calculate the estimated number of cells on the membrane.

### Mammosphere formation assay

MDA-MB-231 and MCF7 cells pretreated with CM were mixed in MammoCult Basal medium (StemCell Technologies Inc., Vancouver, Canada) supplemented with heparin and hydrocortisone to obtain a ratio of 9.5×10^3^ cells/2 ml. This suspension was cultured for 5 days on an ultra-low attachment surface in a 6-well plate (Corning, Corning, NY, USA). The mammosphere (diameter ≥100 µm) forming efficiency (MSFE) was calculated using the following equation:

MSFE (%) = number of mammospheres × 100/number of seeded cells.

### Colony formation assay

MDA-MB-231 and MCF7 cells pretreated with CM were seeded in 6-well plates at a ratio of 100 cells/well. The cells were incubated for 1 week before being fixed with 4% paraformaldehyde and then stained with 5% w/v crystal violet. The colonies were analyzed and counted via macroscopic observations.

### Spheroid culture

MDA-MB-231 cells were treated with M-protein (3 pmol/mL) 48 hours before being collected and stained with PKH26 (Sigma). EPC were collected and stained with CSFE (Dojindo, Japan). Spheroid culture was conducted based on the protocol of Dahndapani et al, 2023 (50), including monoculture, diculture and triculture. Briefly, 100 μL of a mixture of 6x10^4^ cells suspension were seeded into PrimeSurfaceU 96-well plate (Sumibe, Japan). Diculture spheroids consisted of a mixture of MDA-MB-231 cells and EPC with a ratio of 3x10^4^ cells:3x10^4^ cells. Triculture spheroids consisted of a mixture of MDA-MB-231 cells, EPC and ATMSC with a ratio of 2x10^4^ cells:2x10^4^cells:2x10^4^ cells. After 1 week, spheroids were trypsinized to collected single cells. CFSE-labeled EPC were sorted using a flow cytometer (MoFlo XDP, Beckman Coulter) for a further analysis of TEC marker gene expression and tube formation.

### Tube formation assay

EPC and HUVEC were treated for 48 h as described above. Matrigel (300 µL; Corning) was used to coat each well in the 4-well plates and incubated for 30 min before seeding 7.5×10^4^ cells/500 µl medium of the treated EPC and HUVEC. Images of tube formation were taken in nine random areas of the well at 3 h and 6 h after seeding. The lengths of the tubes formed were analyzed using the angiogenesis program in ImageJ.

### Quantitative reverse transcription (qRT) PCR gene expression analyses

ATMSC, EPC, HUVEC, MDA-MB-231 cells, and MCF7 cells were treated with EV or CM as described above. Sepasol-RNA Super G (Nacalai Tesque) was added according to the manufacturer’s instructions to isolate the total RNA, followed by reverse transcription into cDNA using a ReverTra Ace qPCR RT kit (Toyobo, Kita, Osaka, Japan). Two microliters of the cDNA were amplified with the THUNDERBIRD SYB qPCR Mix (Toyobo) via the Real-time PCR system QuantStudio 5 (Thermo Fisher Scientific). The samples were denatured for 10 min at 95°C followed by 15 s cycles of denaturation at 95°C. Finally, 30 s of annealing and extension was performed at 60°C. The level of gene expression in each sample was analyzed using the ΔΔCt method (formula 2^-ΔΔCT^) and normalized to ACTB (β-actin) gene expression. The primers were listed in **Table 1**.

**Table 1:** Primers used for quantitative polymerase chain reaction.

Gene	Forward sequence	Reverse sequence
β-Actin	CTCGCCTTTGCCGATCC	TCTCCATGTCGTCCCAGTTG
FAP	TGTCTGCCAGTCTTCCGTGAAG	GGAAGTGCCTGTTCCAGCAATG
FSP	CAGAACTAAAGGAGCTGCTGACC	CTTGGAAGTCCACCTCGTTGTC
Vimentin	AGGCAAAGCAGGAGTCCACTGA	ATCTGGCGTTCCAGGGACTCAT
IL6	ACAAGAGTAACATGTGTGAAAGCAG	TATACCTCAAACTCCAAAAGACCAG
IL8	GAGAGTGATTGAGAGTGGACCAC	CACAACCCTCTGCACCCAGTTT
CXCL7	CTGGCTTCCTCCACCAAAGG	GACTTGGTTGCAATGGGTTCC
bFGF	CAGAGTGTTGCTGTGACCAG	GATCGAGCTCACTGTGGAGT
PDGFa	ATCAATCAGCCCAGATGGAC	TTCACGGGCAGAAAGGTACT
SDF1	TGAGAGCTCGCTTTGAGTGA	CACCAGGACCTTCTGTGGAT
ANG1	GCCTGATCTTACACGGTGCT	GGCCACAAGCATCAAACCAC
VEGF	CAAGACAAGAAAATCCCTGTGG	CCTCGGCTTGTCACATCTG
IL1α	TGTGACTGCCCAAGATGAAG	AAGTTTGGATGGGCAACTGA
PTGES2	ACCTCTATGAGGCTGCTGACAAGT	CATACACCGCCAAATCAGCGAGAT
COX2	CCCTTGGGTGTCAAAGGTAA	GCCCTCGCTTATGATCTGTC
CXCR7	TCGGCAGCATTTTCTTCCTC	GCAGTCGGTCTCATTGTTGGAC
CXCR4	CCAAGGAAAGCATAGAGGATGGGGTTC	CTGTGACCGCTTCTACCCCAATGACTT
vWF	ATGCCCCTGGAGAAACAGTG	CCGAAAGGTCCCAGGGTTAC
SNAIL	AACTACAGCGAGCTGCAGGACTCTAA	CCTTTCCCACTGTCCTCATCTGACA
VE-Cadherin	CAGAGTACCACCTCACTGCTGTCATT	CCACTGCTGTCACAGAGATGACTGA
ALDH	GGAGTGTTGAGCGGGCTAAGAAGTA	CATTAGAGAACACTGTGGGCTGGAC

### *In vivo* metastasis assay

All experimental procedures were approved by the University of Tsukuba Institute of Animal Care and Use Committee. Female C57BL/6J mice were bred under specific pathogen-free (SPF) conditions. MCF-7 cells after treatment with ATMSC- or EPC-derived CM were collected and resuspended in PBS. Cells with a density of 2×10^5^ cells/300 µl were injected intravenously via the tail vein, followed by a daily injection of cyclosporin-A (Sigma – Aldrich) for the initial week. For the second week, cyclosporine was administered on alternating days. Mice were sacrificed by cervical dislocation after 14 days. The lungs were harvested, fixed with 4% paraformaldehyde (Wako Pure Chemical), frozen, and stained with hematoxylin and eosin. The cross-sections were observed under a microscope using 40x magnification to identify and capture tumor foci. The tumor foci areas are defined as the areas with high cell density, which are dense masses contains cells with epithelial morphology and high nuclear density (purple stained areas), while lung tissue areas have porous structure with low nuclear density (pink stained areas). All sections from one lung sample were observed in the order of cutting, so the tumor foci appeared at the same place in continuous sections would be considered as the same tumors. The size of a certain tumor was defined based on the largest focus of that tumor observed among continuous sections. Images were analyzed using the ImageJ software program.

### Western blotting

Total protein was extracted from EV using RIPA buffer (Nacalai, Kyoto, Japan) and the concentration was measured by a Bradford assay (Bio-Rad). An amount of 50μg of extracted protein was separated by electrophoresis in 7.5% SDS-polyacrylamide gels. The proteins were transferred to a polyvinylidene difluoride membrane (Millipore). After that membranes were blocked with 5% skim milk in Tris-buffered saline containing 0.1% Tween 20 (TBST) for an hour at room temperature. Membranes were then incubated with primary antibodies, including rabbit anti-CD63 (GTX17441; GeneTex), rabbit anti-TSG101 (GTX118736; GeneTex), and rabbit apolipoprotein A1 (APOA1, GTX40453; GeneTex) at 1:1000 dilution overnight at 4°C. Membranes were washed with TBST, then incubated with HRP-conjugated goat anti-rabbit IgG (Thermo Fisher Scientific) at 1:10,000 dilution. The positive signals were analyzed by a luminescence imager (Image Quant LAS4000; GE Health Care, Little Chalfont, United Kingdom) using chemiluminescence reagents (Merck Millipore).

### Statistical analyses

The results are presented as mean± standard deviation (SD). The t-test of the Microsoft Excel software program was used to calculate and analyze differences. P values of<0.05 were considered to indicate statistical significance.

## Data availability statement

The original contributions presented in the study are included in the article/**Supplementary Material**. Further inquiries can be directed to the corresponding author.

## Ethics statement

The studies involving humans were approved by The Ethics Committee of the University of Tsukuba. The studies were conducted in accordance with the local legislation and institutional requirements. The participants provided their written informed consent to participate in this study. The animal study was approved by University of Tsukuba Institute of Animal Care and Use Committee. The study was conducted in accordance with the local legislation and institutional requirements.

## Author contributions

H-NN: Conceptualization, Data curation, Formal Analysis, Investigation, Methodology, Software, Writing – original draft. C-KV: Conceptualization, Data curation, Investigation, Methodology, Project administration, Supervision, Validation, Visualization, Writing – review & editing. MF: Data curation, Formal Analysis, Methodology, Software, Visualization, Writing – review & editing. MU: Data curation, Formal Analysis, Methodology, Validation, Writing – original draft. LT: Data curation, Formal Analysis, Methodology, Software, Writing – original draft. TY: Data curation, Formal Analysis, Methodology, Software, Writing – review & editing. MO-Y: Methodology, Writing – review & editing. HH: Methodology, Writing – review & editing. MO: Methodology, Writing – review & editing. TT: Methodology, Writing – review & editing. YH: Methodology, Project administration, Writing – review & editing. OO: Conceptualization, Data curation, Funding acquisition, Investigation, Project administration, Resources, Supervision, Validation, Visualization, Writing – review & editing.

## Supplementary material

Supplementary Figure 1 Characteristics of EV derived from triple-negative BCC. **(A)** Size distribution of nEV and MpEV. **(B)** The marker expression of nEV and MpEV. **(C)** Uptake of PKH26-labeled EV by ATMSC, examined by flow cytometry.

Supplementary Figure 2 Response of MDA-MB-231 cells to CM derived from EV-ATMSC. **(A)** Migration of MDA-MB-231 cells treated with CM derived from ATMSC, nEV-ATMSC, MpEV-ATMSC. **(B)** Proliferation of MDA-MB-231 cells treated with CM derived from ATMSC, nEV-ATMSC, MpEV-ATMSC. **(C)** Colony formation of MDA-MB-231 cells treated with CM derived from ATMSC, nEV-ATMSC, MpEV-ATMSC. **(D)** Mammosphere formation of MDA-MB-231 cells treated with CM derived from ATMSC, nEV-ATMSC, MpEV-ATMSC.

Supplementary Figure 3 Uptake of nEV and MpEV by EPC, EC, and effects of MpEV secreted from M-protein-induced triple negative BCC on EC. **(A)** Uptake of PKH26-labeled EV by EPC, examined by flow cytometry. **(B)** Uptake of PKH26-labeled EV by EC, examined by flow cytometry. **(C)** Proliferation of HUVEC uptaking EV. **(D)** Transwell migration of HUVEC uptaking EV. **(E)** Tube formation of HUVEC uptaking EV.

Supplementary Figure 4 Response of MDA-MB-231 cells to CM derived from EPC, nEV- EPC, MpEV-EPC. **(A)** Migration of MDA-MB-231 cells treated with CM derived from EPC, nEV- EPC, MpEV-EPC (the scale bars indicate 500 µm). **(B)** Proliferation of MDA-MB-231 cells treated with CM derived from EPC, nEV- EPC, MpEV-EPC. **(C)** Colony formation of MDA-MB-231 cells treated with CM derived from EPC, nEV- EPC, MpEV-EPC. **(D)** Mammosphere formation of MDA-MB-231 cells treated with CM derived from EPC, nEV- EPC, MpEV-EPC.

Supplementary Figure 5 Spheroid culture of PKH26-labeled MDA-MB-231 cells, CFSE-labeled EPC and ATMSC (the scale bars indicate 500 µm, MDA: MDA-MB-231, M-MDA: M-protein-induced MDA-MB-231).

Supplementary Figure 6 Effects of MpEV and nEV on MDA-MB-231 cells. **(A)** Migration of MDA-MB-231 uptaking EV. **(B)** Proliferation of MDA-MB-231 uptaking EV. **(C)** Colony formation of MDA-MB-231 uptaking EV. **(D)** Cytokine gene expression of MDA-MB-231 uptaking EV. **(E)** EMT-related gene expression of MDA-MB-231 uptaking EV.

Supplementary Figure 7 Effects of MpEV on spheroid culture. **(A)** Spheroid culture of PKH26-labeled MDA-MB-231 cells, CFSE-labeled EPC and ATMSC (the scale bars indicate 500 µm). **(B)** TEC-related gene expression of EPC isolated from spheroid. **(C)** Tube formation of EPC isolated from spheroid (The scale bars indicate 500 µm).

## References

[1] Huang X Liang H Zhang H Tian L Cong P Wu T. The potential mechanism of cancer patients appearing more vulnerable to SARS-CoV-2 and poor outcomes: A pan-cancer bioinformatics analysis. Front Immunol. (2021) 12:804387. doi: 10.3389/fimmu.2021.804387 35082790 PMC8784815

[2] Temena MA Acar A. Increased TRIM31 gene expression is positively correlated with SARS-CoV-2 associated genes TMPRSS2 and TMPRSS4 in gastrointestinal cancers. Sci Rep. (2022) 12:11763. doi: 10.1038/s41598-022-15911-2 35970857 PMC9378649

[3] Katopodis P Anikin V Randeva HS Spandidos DA Chatha K Kyrou I. Pan−cancer analysis of transmembrane protease serine 2 and cathepsin L that mediate cellular SARS−CoV−2 infection leading to COVID-19. Int J Oncol. 57(2):533–9. doi: 10.3892/ijo.2020.5071 PMC7307597 32468052

[4] Wei X Su J Yang K Wei J Wan H Cao X. Elevations of serum cancer biomarkers correlate with severity of COVID-19. J Med Virol. 92(10):2036–41. doi: 10.1002/jmv.25957 PMC7267262 32347972

[5] Saygideger Y Sezan A Candevir A Saygıdeğer Demir B Güzel E Baydar O. COVID-19 patients’ sera induce epithelial mesenchymal transition in cancer cells. Cancer Treat Res Commun. (2021) 28:100406. doi: 10.1016/j.ctarc.2021.100406 34090218 PMC8146274

[6] Nguyen H-NT Kawahara M Vuong C-K Fukushige M Yamashita T Ohneda O. SARS-CoV-2 M-protein facilitates Malignant transformation of breast cancer cells. Front Oncol. (2022) 12:923467. doi: 10.3389/fonc.2022.923467 35747796 PMC9209714

[7] Derosa L Melenotte C Griscelli F Gachot B Marabelle A Kroemer G. The immuno-oncological challenge of COVID-19. Nat Cancer. (2020) 1(10):946–64. doi: 10.1038/s43018-020-00122-3 35121872

[8] Turnquist C Ryan BM Horikawa I Harris BT Harris CC. Cytokine storms in cancer and COVID-19. Cancer Cell. (2020) 38:598–601. doi: 10.1016/j.ccell.2020.09.019 33038939 PMC7531591

[9] Zanelli S Fiorio E Zampiva I Zacchi F Borghesani G Giontella E. Risk and severity of SARS-CoV-2 infection in breast cancer patients undergoing a structured infection screening program at the University and Hospital Trust of Verona. Ann oncology : Off J Eur Soc Med Oncol. (2022) 33:661–3. doi: 10.1016/j.annonc.2022.02.227 PMC8904004 35276335

[10] Li J Bai H Qiao H Du C Yao P Zhang Y. Causal effects of COVID-19 on cancer risk: A Mendelian randomization study. J Med Virol. (2023) 95:e28722. doi: 10.1002/jmv.28722 37185860

[11] Vyshnavi AMH Namboori PKK. Association of SARS-coV-2 infection and triple negative breast cancer (TNBC) A computational illustrative study. Lett Drug Design Discovery. (2023) 20:1107–16. doi: 10.2174/1570180819666220620101333

[12] Marenco-Hillembrand L Erben Y Suarez-Meade P Franco-Mesa C Sherman W Eidelman BH. Outcomes and surgical considerations for neurosurgical patients hospitalized with COVID-19–A multicenter case series. World Neurosurg. (2021) 154:e118–29. doi: 10.1016/j.wneu.2021.06.147 PMC8257398 34237448

[13] Bilir C Cakir E Gülbagci B Altindis M Toptan H Guclu E. COVID-19 prevalence and oncologic outcomes of asymptomatic patients with active cancer who received chemotherapy. Acta Med Mediterr. (2021) 37:667–71. doi: 10.19193/0393-6384_2021_1_103

[14] Li T Wang L Wang H Li X Zhang S Xu Y. Serum SARS-COV-2 nucleocapsid protein: A sensitivity and specificity early diagnostic marker for SARS-COV-2 infection. Front Cell Infect Microbiol. (2020) 10:470. doi: 10.3389/fcimb.2020.00470 33014893 PMC7498565

[15] Shan D Johnson JM Fernandes SC Suib H Hwang S Wuelfing D. N-protein presents early in blood, dried blood and saliva during asymptomatic and symptomatic SARS-CoV-2 infection. Nat Commun. (2021) 12(1):1931. doi: 10.1038/s41467-021-22072-9 33771993 PMC7997897

[16] Ogata AF Maley AM Wu C Gilboa T Norman M Lazarovits R. Ultra-sensitive serial profiling of SARS-coV-2 antigens and antibodies in plasma to understand disease progression in COVID-19 patients with severe disease. Clin Chem. (2020) 66(12):1562–72. doi: 10.1093/clinchem/hvaa213 PMC7499543 32897389

[17] Jana AK Greenwood AB Hansmann UHE. Presence of a SARS-COV-2 protein enhances Amyloid Formation of Serum Amyloid A. bioRxiv. (2021) 125(32):9155–9167. doi: 10.1101/2021.05.18.444723v2 PMC8369982 34370466

[18] Sullivan R Maresh G Zhang X Salomon C Hooper J Margolin D. The emerging roles of extracellular vesicles as communication vehicles within the tumor microenvironment and beyond. Front Endocrinol. (2017) 8:194. doi: 10.3389/fendo.2017.00194 PMC5550719 28848498

[19] Baghban R Roshangar L Jahanban-Esfahlan R Seidi K Ebrahimi-Kalan A Jaymand M. Tumor microenvironment complexity and therapeutic implications at a glance. Cell Commun Signal. (2020) 18(1):59. doi: 10.1186/s12964-020-0530-4 32264958 PMC7140346

[20] Wendler F Favicchio R Simon T Alifrangis C Stebbing J Giamas G. Extracellular vesicles swarm the cancer microenvironment: from tumor–stroma communication to drug intervention. Oncogene. (2017) 36:877–84. doi: 10.1038/onc.2016.253 27546617

[21] Cho JA Park H Lim EH Lee KW. Exosomes from breast cancer cells can convert adipose tissue-derived mesenchymal stem cells into myofibroblast-like cells. Int J Oncol. (2012) 40:130–8. doi: 10.3892/ijo.2011.1193 21904773

[22] Escobar P Bouclier C Serret J Bièche I Brigitte M Caicedo A. IL-1β produced by aggressive breast cancer cells is one of the factors that dictate their interactions with mesenchymal stem cells through chemokine production. Oncotarget. (2015) 6(30):29034–47. doi: 10.18632/oncotarget.4732 PMC4745709 26362269

[23] Amorim M Fernandes G Oliveira P Martins-de-Souza D Dias-Neto E Nunes D. The overexpression of a single oncogene (ERBB2/HER2) alters the proteomic landscape of extracellular vesicles. Proteomics. (2014) 14(12):1472–9. doi: 10.1002/pmic.201300485 24733759

[24] Anderson NM Simon MC. The tumor microenvironment. Curr Biol. (2020) 30:R921–5. doi: 10.1016/j.cub.2020.06.081 PMC8194051 32810447

[25] Khanh VC Fukushige M Moriguchi K Yamashita T Osaka M Hiramatsu Y. Type 2 diabetes mellitus induced paracrine effects on breast cancer metastasis through extracellular vesicles derived from human mesenchymal stem cells. Stem Cells Dev. (2020) 29(21):1382–94. doi: 10.1089/scd.2020.0126 32900278

[26] Hass R. Role of MSC in the tumor microenvironment. Cancers (Basel). (2020) 12(8):2107. doi: 10.3390/cancers12082107 32751163 PMC7464647

[27] Mandel K Yang Y Schambach A Glage S Otte A Hass R. Mesenchymal stem cells directly interact with breast cancer cells and promote tumor cell growth *in vitro* and *in vivo* . Stem Cells Dev. (2013) 22:3114–27. doi: 10.1089/scd.2013.0249 23895436

[28] Shi Y Du L Lin L Wang Y. Tumour-associated mesenchymal stem/stromal cells: emerging therapeutic targets. Nat Rev Drug Discovery. (2017) 16:35–52. doi: 10.1038/nrd.2016.193 27811929

[29] Hill BS Pelagalli A Passaro N Zannetti A. Tumor-educated mesenchymal stem cells promote pro-metastatic phenotype. Oncotarget. (2017) 8:73296–311. doi: 10.18632/oncotarget.20265 PMC5641213 29069870

[30] Gehling UM Ergün S Schumacher U Wagener C Pantel K Otte M. *In vitro* differentiation of endothelial cells from AC133-positive progenitor cells. Blood. (2000) 95:3106–12. doi: 10.1182/blood.V95.10.3106 10807776

[31] Zhao X Liu H Li J Liu X. Endothelial progenitor cells promote tumor growth and progression by enhancing new vessel formation (Review). Oncol Lett. (2016) 12(2):793–9. doi: 10.3892/ol.2016.4733 PMC4950911 27446353

[32] Nolan DJ Ciarrocchi A Mellick AS Jaggi JS Bambino K Gupta S. Bone marrow-derived endothelial progenitor cells are a major determinant of nascent tumor neovascularization. Genes Dev. (2007) 21:1546–58. doi: 10.1101/gad.436307 PMC1891431 17575055

[33] Zhang H Chen FI Xu CP Ping YF Wang QL Liang ZQ. Incorporation of endothelial progenitor cells into the neovasculature of Malignant glioma xenograft. J Neurooncol. (2009) 93(2):165–74. doi: 10.1007/s11060-008-9757-4 19052696

[34] Annan DA-M Kikuchi H Maishi N Hida Y Hida K. Tumor endothelial cell-A biological tool for translational cancer research. Int J Mol Sci. (2020) 21(9):3238. doi: 10.3390/ijms21093238 32375250 PMC7247330

[35] Butler JM Kobayashi H Rafii S. Instructive role of the vascular niche in promoting tumour growth and tissue repair by angiocrine factors. Nat Rev Cancer. (2010) 10:138–46. doi: 10.1038/nrc2791 PMC2944775 20094048

[36] Ritter A Kreis N-N Hoock SC Solbach C Louwen F Yuan J. Adipose tissue-derived mesenchymal stromal/stem cells, obesity and the tumor microenvironment of breast cancer. Cancers (Basel). (2022) 14(16):3908. doi: 10.3390/cancers14163908 36010901 PMC9405791

[37] Gentile P. Breast cancer therapy: the potential role of mesenchymal stem cells in translational biomedical research. Biomedicines. (2022) 10(5):1179. doi: 10.3390/biomedicines10051179 35625915 PMC9138371

[38] Jansson S Aaltonen K Bendahl PO Falck AK Karlsson M Pietras K. The PDGF pathway in breast cancer is linked to tumour aggressiveness, triple-negative subtype and early recurrence. Breast Cancer Res Treat. (2018) 169(2):231–41. doi: 10.1007/s10549-018-4664-7 PMC5945746 29380207

[39] Johnston CL Cox HC Gomm JJ Coombes RC. bFGF and aFGF induce membrane ruffling in breast cancer cells but not in normal breast epithelial cells: FGFR-4 involvement. Biochem J. (1995) 306:609–16. doi: 10.1042/bj3060609 PMC1136561 7534069

[40] Fenig E Livnat T Sharkon-Polak S Wasserman L Beery E Lilling G. Basic fibroblast growth factor potentiates cisplatinum-induced cytotoxicity in MCF-7 human breast cancer cells. J Cancer Res Clin Oncol. (1999) 125(10):556–62. doi: 10.1007/s004320050316 PMC12169193 10473868

[41] Ariad S Seymour L Bezwoda WR. Platelet-derived growth factor (PDGF) in plasma of breast cancer patients: Correlation with stage and rate of progression. Breast Cancer Res Treat. (1991) 20(1):11–7. doi: 10.1007/BF01833352 1667486

[42] Kang H Watkins G Parr C Douglas-Jones A Mansel RE Jiang WG. Stromal cell derived factor-1: its influence on invasiveness and migration of breast cancer cells *in vitro*, and its association with prognosis and survival in human breast cancer. Breast Cancer Res. (2005) 7(4):R402–10. doi: 10.1186/bcr1022 PMC1175055 15987445

[43] Li H-J Reinhardt F Herschman HR Weinberg RA. Cancer-stimulated mesenchymal stem cells create a carcinoma stem cell niche via prostaglandin E2 signaling. Cancer Discovery. (2012) 2(9):840–55. doi: 10.1158/2159-8290.CD-12-0101 PMC3833451 22763855

[44] Parker BS Argani P Cook BP Liangfeng H Chartrand SD Zhang M. Alterations in vascular gene expression in invasive breast carcinoma. Cancer Res. (2004) 64:7857–66. doi: 10.1158/0008-5472.CAN-04-1976 15520192

[45] Bhati R Patterson C Livasy CA Fan C Ketelsen D Hu Z. Molecular characterization of human breast tumor vascular cells. Am J Pathol. (2008) 172(5):1381–90. doi: 10.2353/ajpath.2008.070988 PMC2329846 18403594

[46] Hida K Maishi N Akiyama K Ohmura-Kakutani H Torii C Ohga N. Tumor endothelial cells with high aldehyde dehydrogenase activity show drug resistance. Cancer Sci. (2017) 108(2):2195–203. doi: 10.1111/cas.13388 PMC5666026 28851003

[47] Grange C Bussolati B Bruno S Fonsato V Sapino A Camussi G. Isolation and characterization of human breast tumor-derived endothelial cells. Oncol Rep. (2006) 15(2):381–6. doi: 10.3892/or.15.2.381 16391858

[48] Dhami SPS Patmore S Comerford C Byrne CM Cavanagh B Castle J. Breast cancer cells mediate endothelial cell activation, promoting von Willebrand factor release, tumor adhesion, and transendothelial migration. J Thromb Haemost. (2022) 20(10):2350–65. doi: 10.1111/jth.15794 PMC9796425 35722954

[49] DeCicco-Skinner KL Henry GH Cataisson C Tabib T Gwilliam JC Watson NJ. Endothelial cell tube formation assay for the *in vitro* study of angiogenesis. J Vis Exp: JoVE. (2014) 91:e51312. doi: 10.3791/51312 PMC4540586 25225985

[50] Dhandapani H Siddiqui A Karadkar S Tayalia P. *In vitro* 3D spheroid model preserves tumor microenvironment of hot and cold breast cancer subtypes. Adv Healthc. Mater. (2023) 12(21):e2300164. doi: 10.1002/adhm.202300164 37141121

[51] Stavri GT Zachary IC Baskerville PA Martin JF Erusalimsky JD. Basic fibroblast growth factor upregulates the expression of vascular endothelial growth factor in vascular smooth muscle cells. Synergistic interaction with hypoxia. Circulation. (1995) 92(1):11–4. doi: 10.1161/01.cir.92.1.11 7788904

[52] Mirshahi F Pourtau J Li H Muraine M Trochon V Legrand E. SDF-1 activity on microvascular endothelial cells: consequences on angiogenesis in *in vitro* and *in vivo* models. Thromb Res. (2000) 99(6):587–94. doi: 10.1016/s0049-3848(00)00292-9 10974345

[53] Heidemann J Ogawa H Dwinell MB Rafiee P Maaser C Gockel HR. Angiogenic effects of interleukin 8 (CXCL8) in human intestinal microvascular endothelial cells are mediated by CXCR2. J Biol Chem. (2003) 278(10):8508–15. doi: 10.1074/jbc.M208231200 12496258

[54] Bertuzzi AF Marrari A Gennaro N Cariboni U Ciccarelli M Giordano L. Low incidence of sars-cov-2 in patients with solid tumours on active treatment: An observational study at a tertiary cancer centre in lombardy, Italy. Cancers (Basel). (2020) 12(9):1–9. doi: 10.3390/cancers12092352 PMC7564537 32825295

[55] de Joode K Oostvogels AAM GeurtsvanKessel CH de Vries RD Mathijssen RHJ Debets R. Case report: adequate T and B cell responses in a SARS-coV-2 infected patient after immune checkpoint inhibition. Front Immunol. (2021) 12:627186. doi: 10.3389/fimmu.2021.627186 33613575 PMC7889602

[56] Malkani N Rashid MU. SARS-COV-2 infection and lung tumor microenvironment. Mol Biol Rep. (2021) 48(2):1925–34. doi: 10.1007/s11033-021-06149-8 PMC7826145 33486674

[57] Giurini EF Williams M Morin A Zloza A Gupta KH. Inactivated SARS-coV-2 reprograms the tumor immune microenvironment and improves murine cancer outcomes. bioRxiv. (2022) 2022.06.30.498305. doi: 10.1101/2022.06.30.498305

[58] Zhang Z Nomura N Muramoto Y Ekimoto T Uemura T Liu K. Structure of SARS-CoV-2 membrane protein essential for virus assembly. Nat Commun. (2022) 13(1):4399. doi: 10.1038/s41467-022-32019-3 35931673 PMC9355944

[59] Evdokimova V Tognon C Ng T Sorensen PHB. Reduced proliferation and enhanced migration: two sides of the same coin? Molecular mechanisms of metastatic progression by YB-1. Cell Cycle. (2009) 8:2901–6. doi: 10.4161/cc.8.18.9537 19713745

[60] Zhan Q Liu B Situ X Luo Y Fu T Wang Y. New insights into the correlations between circulating tumor cells and target organ metastasis. Signal Transduction Targeting Ther. (2023) 8(1):465. doi: 10.1038/s41392-023-01725-9 PMC10739776 38129401

[61] Xuan X Tian C Zhao M Sun Y Huang C. Mesenchymal stem cells in cancer progression and anticancer therapeutic resistance. Cancer Cell Int. (2021) 21:595. doi: 10.1186/s12935-021-02300-4 34736460 PMC8570012

[62] Zhao W Wang C Liu R Wei C Duan J Liu K. Effect of TGF-β1 on the migration and recruitment of mesenchymal stem cells after vascular balloon injury: involvement of matrix metalloproteinase-14. Sci Rep. (2016) 6(1):21176. doi: 10.1038/srep21176 26880204 PMC4754777

[63] Ridge SM Sullivan FJ Glynn SA. Mesenchymal stem cells: key players in cancer progression. Mol Cancer. (2017) 16:31. doi: 10.1186/s12943-017-0597-8 28148268 PMC5286812

[64] Hossain A Gumin J Gao F Figueroa J Shinojima N Takezaki T. Mesenchymal stem cells isolated from human gliomas increase proliferation and maintain stemness of glioma stem cells through the IL-6/gp130/STAT3 pathway. Stem Cells. (2015) 33(8):2400–15. doi: 10.1002/stem.2053 PMC4509942 25966666

[65] Kansy BA Dißmann PA Hemeda H Bruderek K Westerkamp AM Jagalski V. The bidirectional tumor–mesenchymal stromal cell interaction promotes the progression of head and neck cancer. Stem Cell Res Ther. (2014) 5(4):95. doi: 10.1186/scrt484 25115189 PMC4535379

[66] McLean K Gong Y Choi Y Deng N Yang K Bai S. Human ovarian carcinoma–associated mesenchymal stem cells regulate cancer stem cells and tumorigenesis via altered BMP production. J Clin Invest. (2011) 121:3206–19. doi: 10.1172/JCI45273 PMC3148732 21737876

[67] Kim E-K Kim HJ Yang YI Kim JT Choi MY Choi CS. Endogenous gastric-resident mesenchymal stem cells contribute to formation of cancer stroma and progression of gastric cancer. Korean J Pathol. (2013) 47(6):507–18. doi: 10.4132/KoreanJPathol.2013.47.6.507 PMC3887152 24421843

[68] Li W Zhou Y Yang J Zhang X Zhang H Zhang T. Gastric cancer-derived mesenchymal stem cells prompt gastric cancer progression through secretion of interleukin-8. J Exp Clin Cancer Res. (2015) 34(1):52. doi: 10.1186/s13046-015-0172-3 25986392 PMC4443537

[69] Langer HF Stellos K Steingen C Froihofer A Schönberger T Krämer B. Platelet derived bFGF mediates vascular integrative mechanisms of mesenchymal stem cells *in vitro* . J Mol Cell Cardiol. (2009) 47(2):315–25. doi: 10.1016/j.yjmcc.2009.03.011 19328809

[70] Ball SG Shuttleworth CA Kielty CM. Mesenchymal stem cells and neovascularization: role of platelet-derived growth factor receptors. J Cell Mol Med. (2007) 11(5):1012–30. doi: 10.1111/j.1582-4934.2007.00120.x PMC4401270 17979880

[71] Roccaro AM Sacco A Maiso P Azab AK Tai YT Reagan M. BM mesenchymal stromal cell-derived exosomes facilitate multiple myeloma progression. J Clin Invest. (2013) 123(4):1542–55. doi: 10.1172/JCI66517 PMC3613927 23454749

[72] Asahara T Murohara T Sullivan A Silver M van der Zee R Li T. Isolation of putative progenitor endothelial cells for angiogenesis. Sci (80-.). (1997) 275(5302):964–6. doi: 10.1126/science.275.5302.964 9020076

[73] Peichev M Naiyer AJ Pereira D Zhu Z Lane WJ Williams M. Expression of VEGFR-2 and AC133 by circulating human CD34+ cells identifies a population of functional endothelial precursors. Blood. (2000) 95(3):952–8. doi: 10.1182/blood.V95.3.952.003k27_952_958 10648408

[74] Bussolati B Deregibus M Camussi G. Characterization of molecular and functional alterations of tumor endothelial cells to design anti-angiogenic strategies. Curr Vasc Pharmacol. (2010) 8:220–32. doi: 10.2174/157016110790887036 19485921

[75] Hida K Ohga N Akiyama K Maishi N Hida Y. Heterogeneity of tumor endothelial cells. Cancer Sci. (2013) 104(11):1391–5. doi: 10.1111/cas.12251 PMC7654244 23930621

[76] Pirtskhalaishvili G Nelson JB. Endothelium-derived factors as paracrine mediators of prostate cancer progression. Prostate. (2000) 44(1):77–87. doi: 10.1002/1097-0045(20000615 10861760

[77] Marçola M Rodrigues CE. Endothelial progenitor cells in tumor angiogenesis: another brick in the wall. Stem Cells Int. (2015) 2015:832649. doi: 10.1155/2015/832649 26000021 PMC4427119

[78] Salazar N Zabel BA. Support of tumor endothelial cells by chemokine receptors. Front Immunol. (2019) 10:147. doi: 10.3389/fimmu.2019.00147 30800123 PMC6375834

[79] Zabel BA Wang Y Lewén S Berahovich RD Penfold MET Zhang P. Elucidation of CXCR7-mediated signaling events and inhibition of CXCR4-mediated tumor cell transendothelial migration by CXCR7 ligands. J Immunol. (2009) 183(5):3204–11. doi: 10.4049/jimmunol.0900269 19641136

[80] Beckermann BM Kallifatidis G Groth A Frommhold D Apel A Mattern J. VEGF expression by mesenchymal stem cells contributes to angiogenesis in pancreatic carcinoma. Br J Cancer. (2008) 99(4):622–31. doi: 10.1038/sj.bjc.6604508 PMC2527820 18665180

[81] Orimo A Gupta PB Sgroi DC Arenzana-Seisdedos F Delaunay T Naeem R. Stromal fibroblasts present in invasive human breast carcinomas promote tumor growth and angiogenesis through elevated SDF-1/CXCL12 secretion. Cell. (2005) 121(3):335–48. doi: 10.1016/j.cell.2005.02.034 15882617

[82] Kornilov R Puhka M Mannerström B Hiidenmaa H Peltoniemi H Siljander P. Efficient ultrafiltration-based protocol to deplete extracellular vesicles from fetal bovine serum. J Extracell. vesicles. (2018) 7(1):1422674. doi: 10.1080/20013078.2017.1422674 29410778 PMC5795649

[83] Kimura K Nagano M Salazar G Yamashita T Tsuboi I Mishima H. The role of CCL5 in the ability of adipose tissue-derived mesenchymal stem cells to support repair of ischemic regions. Stem Cells Dev. (2014) 23(5):488–501. doi: 10.1089/scd.2013.0307 24171667 PMC3928761

[84] Nagano M Yamashita T Hamada H Ohneda K Kimura KI Nakagawa T. Identification of functional endothelial progenitor cells suitable for the treatment of ischemic tissue using human umbilical cord blood. Blood. (2007) 110(1):151–60. doi: 10.1182/blood-2006-10-047092 17379743

