# Supplementary figures and images for: Extracellular vesicles derived from SARS-CoV-2 M-protein-induced triple negative breast cancer cells promoted the ability of tissue stem cells supporting cancer progression
Source: Front Oncol. 2024 Mar 7;14:1346312. doi: 10.3389/fonc.2024.1346312 (PMC10955079; doi:10.3389/fonc.2024.1346312)

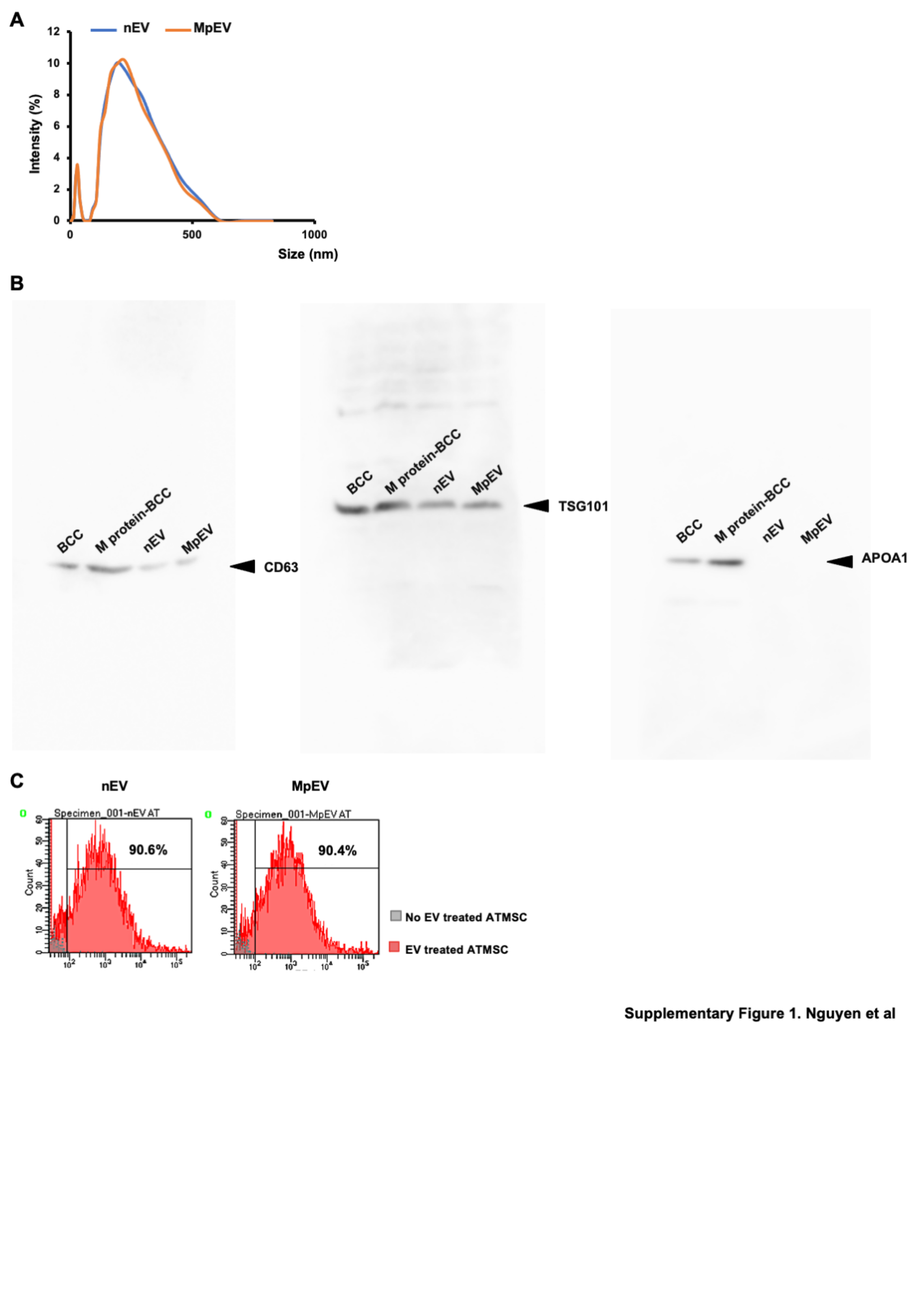

Supplement: Supplementary Figure 1 — Characteristics of EV derived from triple-negative BCC. (A) Size distribution of nEV and MpEV. (B) The marker expression of nEV and MpEV. (C) Uptake of PKH26-labeled EV by ATMSC, examined by flow cytometry. [file Image_1.tiff]

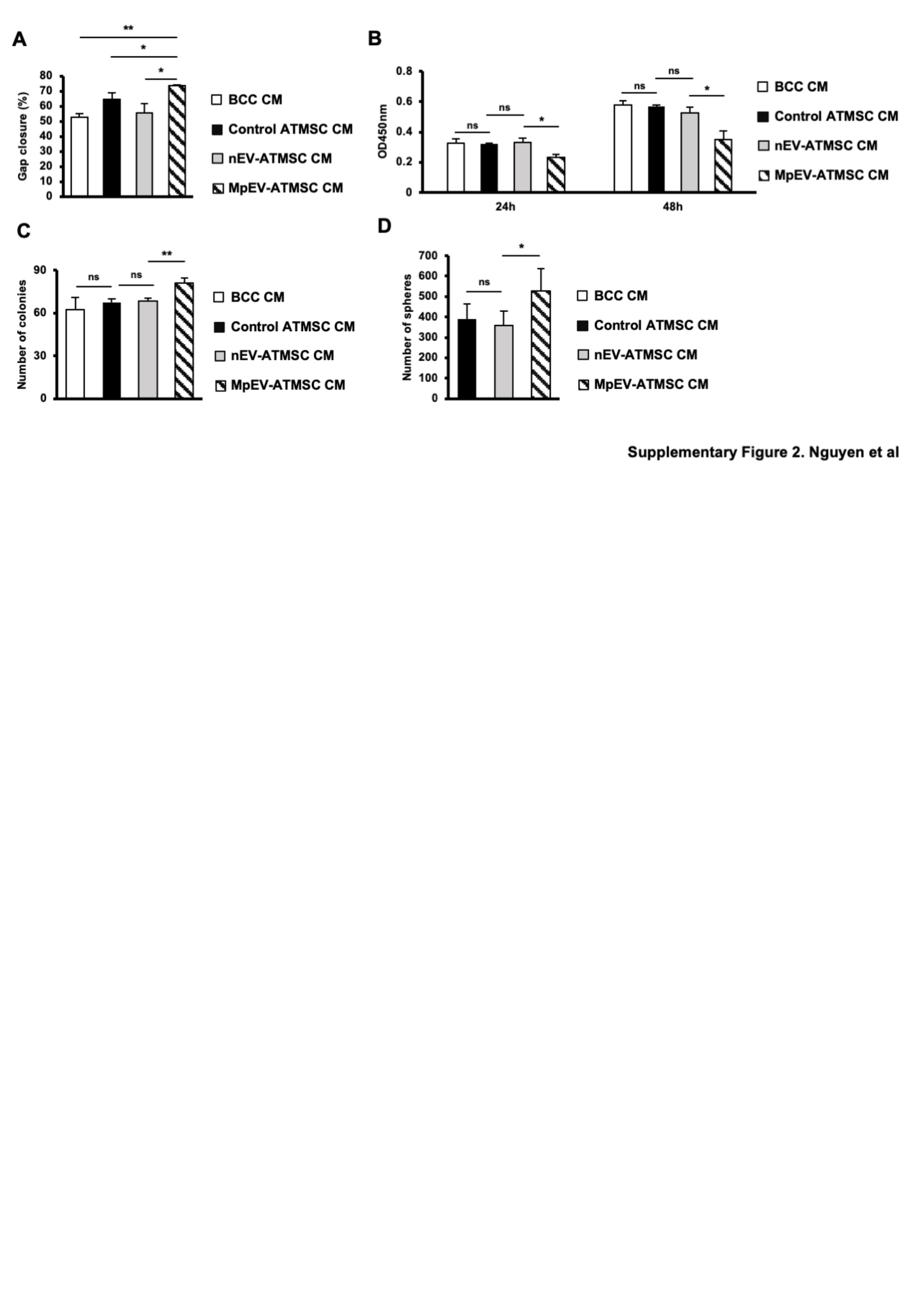

Supplement: Supplementary Figure 2 — Response of MDA-MB-231 cells to CM derived from EV-ATMSC. (A) Migration of MDA-MB-231 cells treated with CM derived from ATMSC, nEV-ATMSC, MpEV-ATMSC. (B) Proliferation of MDA-MB-231 cells treated with CM derived from ATMSC, nEV-ATMSC, MpEV-ATMSC. (C) Colony formation of MDA-MB-231 cells treated with CM derived from ATMSC, nEV-ATMSC, MpEV-ATMSC. (D) Mammosphere formation of MDA-MB-231 cells treated with CM derived from ATMSC, nEV-ATMSC, MpEV-ATMSC. [file Image_2.tiff]

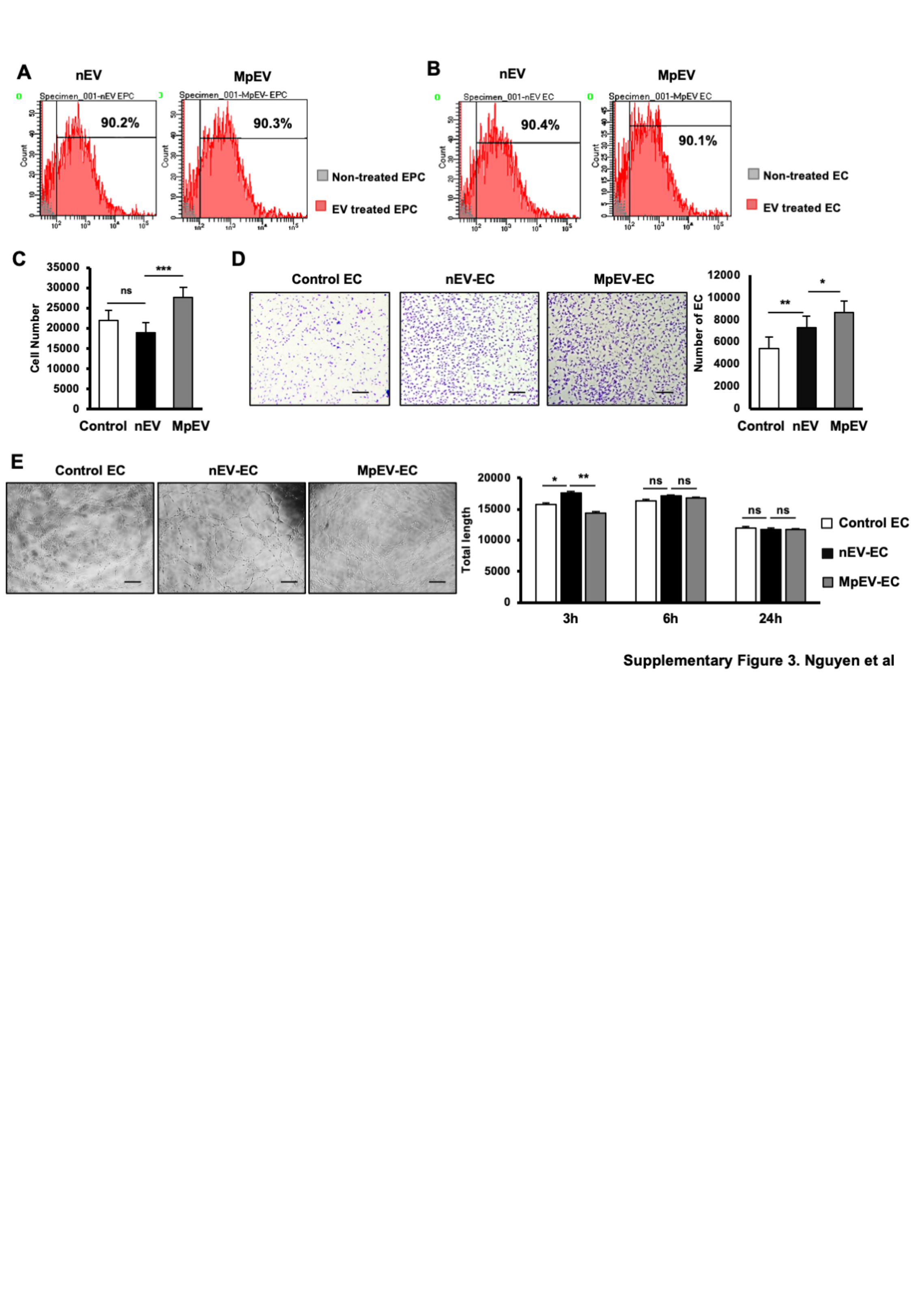

Supplement: Supplementary Figure 3 — Uptake of nEV and MpEV by EPC, EC, and effects of MpEV secreted from M-protein-induced triple negative BCC on EC. (A) Uptake of PKH26-labeled EV by EPC, examined by flow cytometry. (B) Uptake of PKH26-labeled EV by EC, examined by flow cytometry. (C) Proliferation of HUVEC uptaking EV. (D) Transwell migration of HUVEC uptaking EV. (E) Tube formation of HUVEC uptaking EV. [file Image_3.tiff]

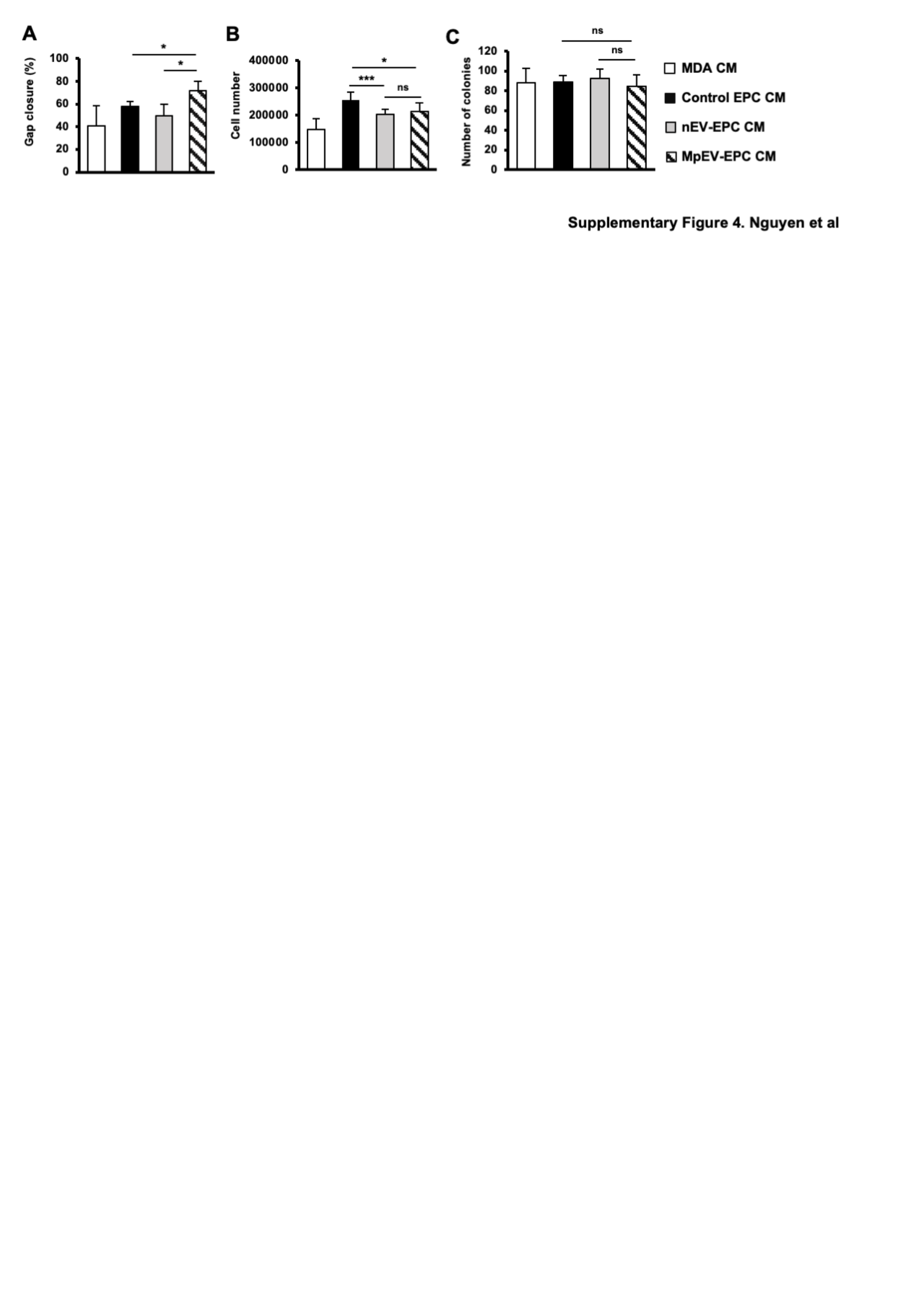

Supplement: Supplementary Figure 4 — Response of MDA-MB-231 cells to CM derived from EPC, nEV- EPC, MpEV-EPC. (A) Migration of MDA-MB-231 cells treated with CM derived from EPC, nEV- EPC, MpEV-EPC (the scale bars indicate 500 µm). (B) Proliferation of MDA-MB-231 cells treated with CM derived from EPC, nEV- EPC, MpEV-EPC. (C) Colony formation of MDA-MB-231 cells treated with CM derived from EPC, nEV- EPC, MpEV-EPC. (D) Mammosphere formation of MDA-MB-231 cells treated with CM derived from EPC, nEV- EPC, MpEV-EPC. [file Image_4.tiff]

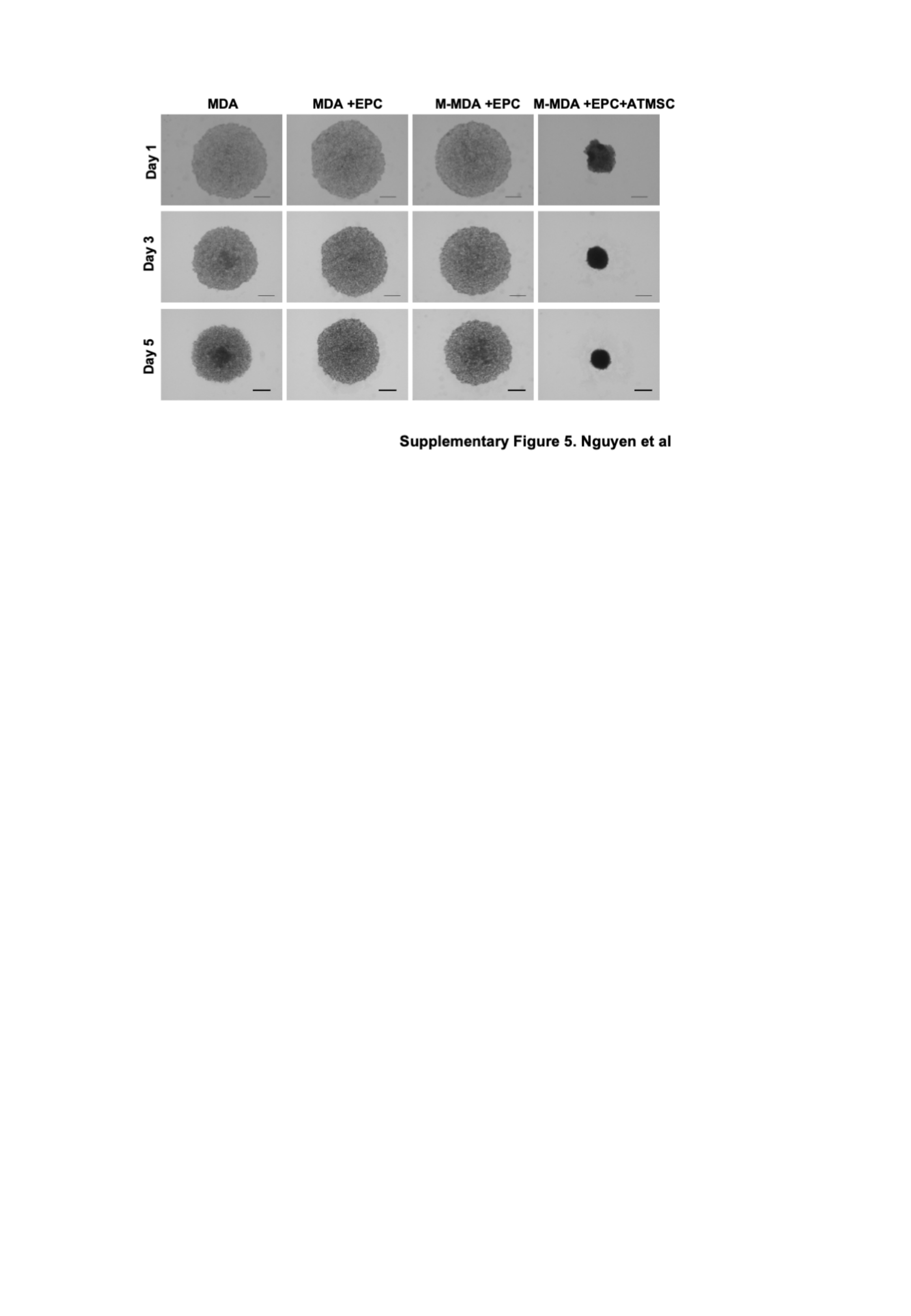

Supplement: Supplementary Figure 5 — Spheroid culture of PKH26-labeled MDA-MB-231 cells, CFSE-labeled EPC and ATMSC (the scale bars indicate 500 µm, MDA: MDA-MB-231, M-MDA: M-protein-induced MDA-MB-231). [file Image_5.tiff]

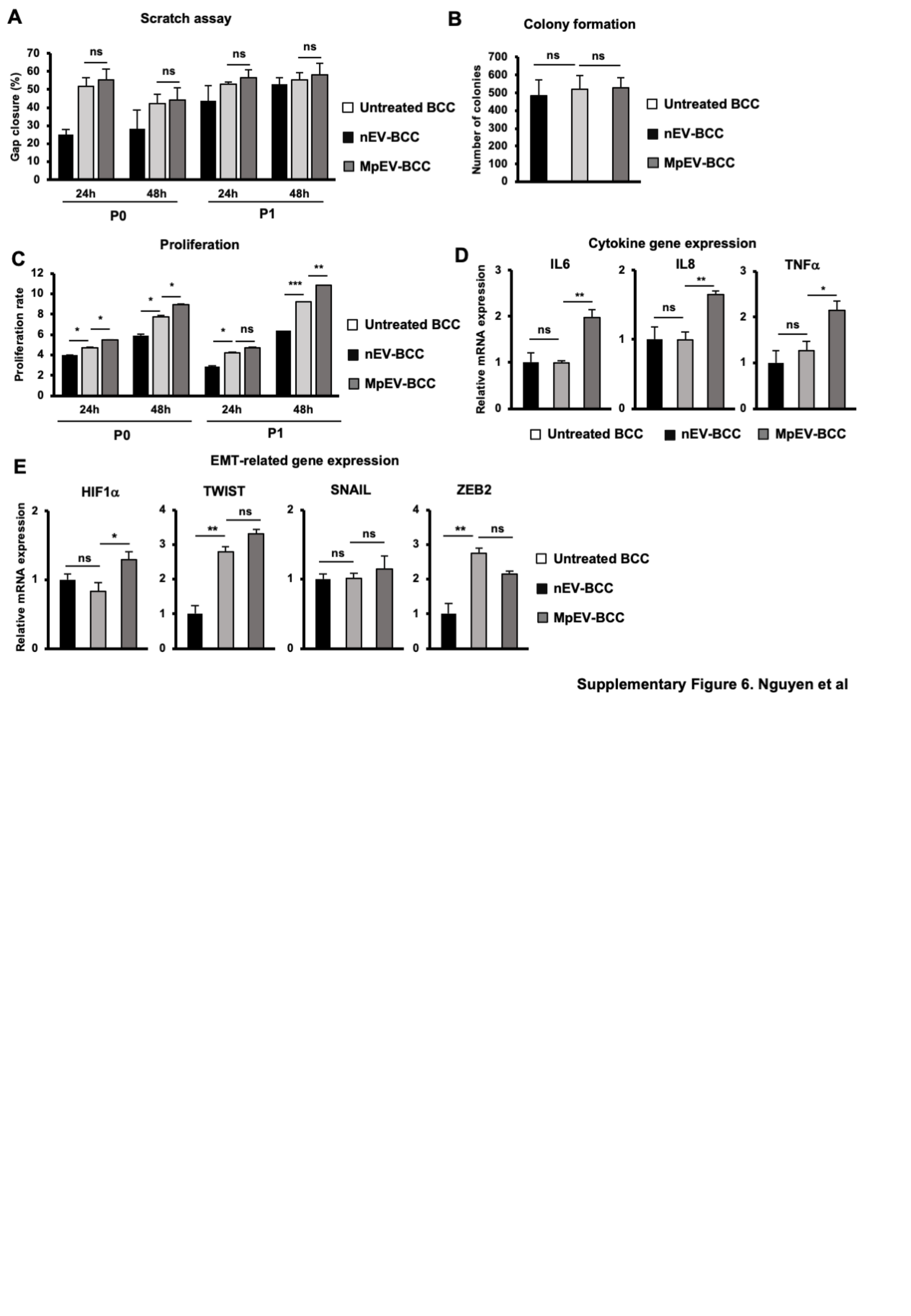

Supplement: Supplementary Figure 6 — Effects of MpEV and nEV on MDA-MB-231 cells. (A) Migration of MDA-MB-231 uptaking EV. (B) Proliferation of MDA-MB-231 uptaking EV. (C) Colony formation of MDA-MB-231 uptaking EV. (D) Cytokine gene expression of MDA-MB-231 uptaking EV. (E) EMT-related gene expression of MDA-MB-231 uptaking EV. [file Image_6.tiff]

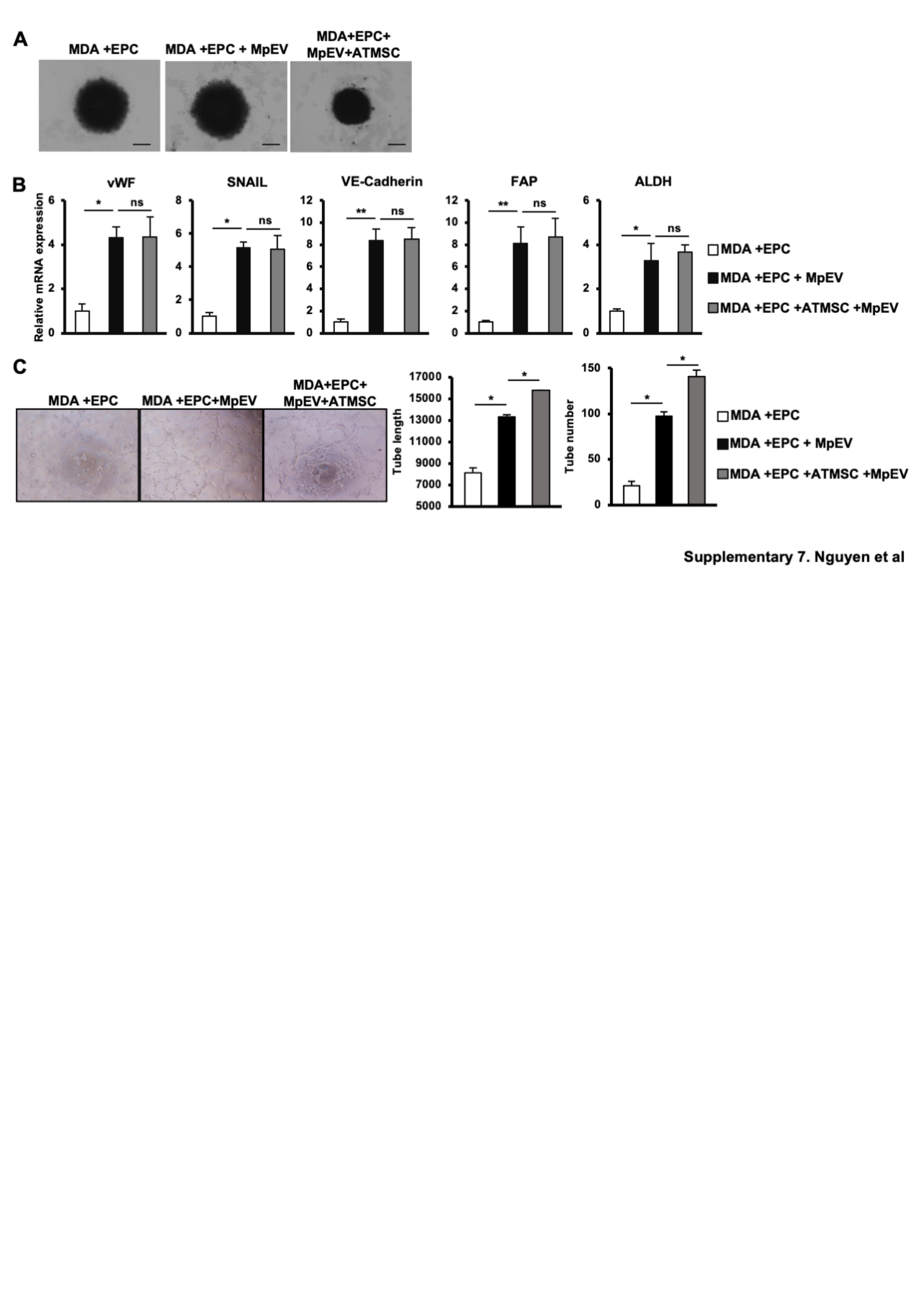

Supplement: Supplementary Figure 7 — Effects of MpEV on spheroid culture. (A) Spheroid culture of PKH26-labeled MDA-MB-231 cells, CFSE-labeled EPC and ATMSC (the scale bars indicate 500 µm). (B) TEC-related gene expression of EPC isolated from spheroid. (C) Tube formation of EPC isolated from spheroid (The scale bars indicate 500 µm). [file Image_7.tiff]
